# Portraying the ‘Chinese international students’: a review of English-language and Chinese-language literature on Chinese international students (2015–2020)

**DOI:** 10.1007/s12564-021-09731-8

**Published:** 2021-11-27

**Authors:** Cora Lingling Xu

**Affiliations:** School of Education, Durham University, Durham, DH1 1TA UK

**Keywords:** Chinese international students, Epistemic justice, Subject position, Post-colonial

## Abstract

The Chinese international students are often portrayed in a monolithic manner in popular discourse. To offer a more comprehensive and critical representation of the Chinese international students, this paper conducts a thematic narrative review of 128 English-language and 74 Chinese-language peer-reviewed articles published between 2015 and 2020. Drawing on post-colonial theories, this review identifies four subject positions portrayed of the Chinese international students: the (1) neoliberal, (2) political, (3) pedagogic and (4) racialised subjects. This paper celebrates heartening developments in the literature which affirms Chinese international students’ epistemic contributions, legitimate pedagogic needs, notable heterogeneity and wide-ranging political, cultural and pedagogic agencies. It also highlights how aspects of these subject positions have exercised epistemic injustice on the Chinese international students. Meanwhile, it pinpoints the Chinese international students’ acquiescence in exacerbating global education inequalities. Among the first to bring the dominant English-language and ‘local’ perspectives of Chinese-language literature in dialogue, this article notes divergent focuses and indicates unique contributions to historicising research on Chinese international students made by the latter. This article challenges popular perceptions of Chinese international students, questions production of knowledge, and pinpoints future research directions.

**Supplementary Information:**

The online version contains supplementary material available at 10.1007/s12564-021-09731-8.

## Introduction

The Chinese international students^1^ have often been understood as monolithic, whether in state policies, media discourse or scholarly discussions (Song, 2019; Sude et al., 2020). As a major source of knowledge production about the Chinese international students, scholarly representations of the Chinese international students occupy a pivotal position in shaping public views of the Chinese international students (Moosavi, 2021). To date, wide-ranging scholarship on Chinese international students has mushroomed across the cogent fields of education, sociology, human geography, anthropology, psychology, tourism, business, and marketing research. Integrating these disparate bodies of academic research to focus the field and make further progress seems pivotal. Moreover, while much of this literature is published in English language, a substantial body of academic research has been published in these students’ native Chinese language, which has received little international attention (Yang, 2018).

In view of these gaps, in this article, I have conducted a thematic narrative review (Lunsford et al., 2016) of 128 English-language and 74 Chinese-language peer-reviewed journal articles published between 2015 and 2020. I offer a critique on how the Chinese international students are portrayed by drawing on post-colonial theories (Bhambra, 2014; Said, 2003 [1978]; Song, 2019; Suspitsyna & Shalka, 2019). I argue that the Chinese international students have been accorded four subject positions, including the (1) neoliberal, (2) political, (3) pedagogic and (4) racialised subjects. I will highlight the heartening nascent research that accentuates Chinese international students’ epistemic contributions, legitimate pedagogic needs, notable heterogeneity and wide-ranging political, cultural and pedagogic agencies, while challenging systemic inequalities imposed on them. Meanwhile, I will discuss how aspects of these subject positions have exercised epistemic injustice on them. I will also pinpoint the Chinese international students’ acquiescence in this process.

As such, this article aims to make three key contributions. Firstly, by bridging the knowledge gap between the dominant English-language literature and the ‘local’ perspectives of Chinese-language authors, this paper challenges the hierarchies of knowledge production (to be elaborated) and affirms the strengths, legitimacy and contributions of the Chinese-language literature. Secondly, by critiquing how Chinese international students are portrayed in both English-language and Chinese-language literature through a post-colonial lens, this article strives to unearth problematic representations that have led to the systemic marginalisation of these students. Such a critique will serve as salient evidence and means to interrogate power hierarchies and the normalisation of western-centric assumptions and prescriptions; this will also constitute a first step to placing Chinese international students as epistemic equals to their western counterparts. Thirdly, through comparing the divergent focuses and approaches of the English- and Chinese-language literature in contexts, this article pinpoints the respective strengths and limitations of each body of literature, thus critically identifying pivotal gaps and future directions for research on Chinese international students.

### Literature gaps

There have been three seminal reviews about research on or media representations of the Chinese international students. Henze and Zhu’s (2012, p. 91) review focussed primarily on the Chinese international students’ pedagogic positions as represented in scholarly work published by 2011. They accentuated the ‘clarification of [Chinese international students] as a source of “irritation” and as a challenge and option for mutual learning’. Suspitsyna and Shalka (2019) reviewed media representation of the Chinese international students in the Chronicle of Higher Education between 2011 and 2015. They pinpointed the harmful effects that colonial media representations of the Chinese international students can have on to campus climate, culture and internationalisation of American campuses. Similarly, Zhang-Wu’s (2018) landscape review of 21 studies about Chinese international students’ experiences in American higher education (HE hereafter) published between 2000 and 2016 revealed colour-blind racism across the literature and had a sole focus on the US context. All three review studies focussed on English-language literature only. These characteristics necessitate a more updated (2015–2020) and comprehensive review of both English- and Chinese-language literature–what this article strives to achieve. In what follows, I will discuss the rationale behind reviewing both the English-language and the Chinese-language literature and the reasons for examining the Chinese international students’ subject positions portrayed.

### Why review the Chinese-language literature? A post-colonial theoretical lens

While there is an abundance of English-language literature on Chinese international students, little is known internationally about what topics of interest and research agendas are for authors of the Chinese-language literature. This gap in knowledge could be placed within global scales of knowledge and power inequalities, both historically and contemporarily. Said’s (2003 [1978]) *Orientalism* provides fundamental understandings about how the Occident/West constructs an Orient/East as the ‘other’ or ‘others’. This is closely related to how the Chinese international students are portrayed in Western-based media and literature (Song, 2019). *Orientalism* is characterised by a complicated and invested system of theory and practice, sedimented through many generations, ‘filter through the Orient into Western consciousness’ (Said, 2003 [1978], p. 6). This feature of *Orientalism* can be, as I shall demonstrate shortly through the Chinese-language literature’s historical studies of the Chinese international students, closely corroborated regarding Western material and ideological investments on generations of Chinese international students. Throughout the Chinese international students’ history, a western-dominant hegemony, i.e. ‘a form of cultural leadership and domination/consent’ (ibid., 7), has been reinforced.

Such a cultural hegemony dictates the relationship between the non-Western individuals (e.g. the Chinese international students) and their Western counterparts. Bhabha’s notion of mimicry (Suspitsyna & Shalka, 2019, p. 291), for instance, depicts how the non-western individual is considered as epistemically inferior and needing work (e.g. pedagogic efforts) to ‘display the appearance and manners of the colonisers’ (ibid.). However, they are never good enough because their mimicry is always ‘partial, contradictory, and ambivalent’ (ibid.). The concept of mimicry thus serves as a useful lens to examine the self-contradictory subject positions of the Chinese international students as pedagogic and racialised subjects, to be elaborated.

In academic research and publishing, the dominance of the West and the ‘othering’ of the Orient have manifest through the privileging of the English-language literature and marginalisation of the ‘local’ or ‘indigenous’ (e.g. Chinese) language literature (Yang, 2018, p. 47). Such geopolitics of knowledge production thus creates hierarchies of knowledge, practices and perspectives, with some being consecrated as legitimate and concomitantly normalised, while others deemed as irrelevant and dismissed (Bhambra, 2014). Moreover, English-language dominance in academic publishing can create ‘subject positions that contribute to their own subjectification’ (Suspitsyna & Shalka, 2019, p. 291). The sorting and ordering of subject positions according to such sets of logics can have pernicious effects, producing subjectivities as more or less powerful, legitimate, or deserving. Thus, it will be a critical way for this article to reveal hidden inequalities and challenge global power hierarchies through unpacking how Chinese international students are ‘portrayed’ in research, and unravelling the kinds of positioning or subject positions that they are rendered, thereby placing these in the broader context of hierarchies of subject positions.

Moreover, this paper’s endeavour to bring in the Chinese-language literature on Chinese international students can be considered as an attempt to bridge the knowledge gap between the dominant English-language literature and the ‘local’ perspectives of Chinese-language authors. In so doing, it also challenges the above-mentioned hierarchies of knowledge production, through affirming the legitimacy, relevance and contributions of the Chinese-language literature. This is, however, not to be mistaken as a blanket endorsement of the Chinese-language literature. Instead, this paper recognises that knowledge production in China (from Qing Dynasty through to contemporary People’s Republic of China era) is shaped by the country’s social and political contexts (Chou, 2014; Guiheux & Simeng, 2018; Miao & Huang, 2020). This means that certain topics and ideological orientations can be safer to adopt and to publish in Chinese-language, but not others. Similarly, in English-language literature, globalisation and academic capitalism (Su & Harrison, 2016) may imply that certain discursive orientations (e.g. of international students as neoliberal subjects) fit political agenda and policy climates of (western) host countries more than others (Song, 2019). As I will demonstrate, this paper will offer critical reviews on both bodies of literature in relation to how they have portrayed (or not) aspects of the Chinese international students.

### Why study the Chinese international students’ portrayed subject positions?

In this article, subject positions refer to socially constructed subjectivities assigned to the Chinese international students within the two bodies of literature reviewed. This article takes the view that academic research and the discursive work done within research can be construed as technologies, in a Foucauldian sense, through which the Chinese international students as subjects are created. In academic research, subject positions can be produced, negotiated and transformed through discursive practices (Raaper, 2020, p. 145); such subject positions generated to represent the researched (i.e. the Chinese international students) can have far-reaching effects and implications such as reinforcing reified status and stereotypes against the researched.

Indeed, there has been an emerging consensus that the Chinese international students have often been uncritically portrayed as ‘an undifferentiated monolith’ (Sude et al., 2020, p. 2), with little regard to their economic, political, ethnic, epistemic backgrounds and beliefs. They are often depicted in ‘limited, static and sometimes implicitly negative’ (ibid, 1) and ‘lop-sided’ ways (Heng, 2020, p. 540) with an overemphasis on the permanent nature of the challenges they experience. In view of this, some researchers call for ‘scholars to be transparent about and revise their ideological constructions of the [Chinese international students]’ (Sude et al., 2020, p. 1).

Another notable trend in popular understanding of the Chinese international students has been an ‘instrumentalist’ mentality and approach which revolve around the Chinese international students’ ‘achievement and accumulations’ (Wu & Tarc, 2019, p. 16). Instead, Wu and Tarc called for ‘deeper, more compelling and existentially significant ways’ of engaging with the Chinese international students’ transnational experiences (ibid.).

Aligned with these critiques from the latest scholarship on the Chinese international students, this article presents an attempt to expose not only Western scholars’, but also Chinese scholars’ ‘ideological constructions’ of the Chinese international students. This underpins the method of documenting authors’ ethnic backgrounds and where they received their research training (to be elaborated). It is hoped that this paper can present a more comprehensive range of subject positions of the Chinese international students, point out current omissions and signpost future research directions.

## Method

For the English-language literature, I chose Google Scholar as the database because of its proven comprehensive coverage of scholarly literature in social sciences, which is often insufficiently indexed in other well-known databases such as Web of Science or Scopus (Martín-Martín et al., 2018). I conducted keyword searches using ‘Chinese international student*^2^’, ‘Chinese overseas student*’, ‘Chinese student*’ AND ‘abroad’ for articles published between 2015 and 2020. The searches yielded 747 results of academic publications—out of these, 298 were considered relevant because they dealt with Chinese international students as substantial research subjects^3^; these were exported to a designated section in my EndNote library. I then eliminated entries that were either repetitive or deemed as inappropriate for this review. These exclusion criteria included (1) student symposia and conference papers (*N* = 12); (2) dissertations (*N* = 142); (3) published in Korean language (*N* = 3); (4) book chapters (*N* = 12); (5) about international students in general but not specifically about Chinese international students (*N* = 4); (6) editorial commentaries (*N* = 1); (7) about international students’ experiences in China (*N* = 5); (8) about Chinese visiting scholars abroad (*N* = 2); (9) were about mainland Chinese students in Hong Kong, but not abroad (*N* = 1). After this round of elimination, the library contains 128 English-language articles for final analysis.

For the Chinese-language literature, I conducted keyword searches on the China Academic Journals full-text database (also known as CNKI, https://www.cnki.net/), which is the largest and continuously updated Chinese journals database in the world. These searches included combinations of ‘中国留学生 (*zhonguo liuxusheng,* Chinese international students)’ Not ‘来华 (*laihua*, coming to China)’, published between 2015 and 2020. I selected publications included within SCI 来源期刊 (*laiyuan qikan*, SCI-origin journals), 核心期刊 (*hexin qikan*, core journals), and CSSCI.^4^ These searches yielded 129 results and I selected 90 relevant entries (based on their research focuses) to import to my EndNote library. Then I engaged with the entries’ abstracts and main body of texts and eliminated articles based on these criteria: (1) about international students in China (*N* = 5); (2) book reviews (*N* = 1); (3) published in non-peer-reviewed journals (*N* = 8); (4) about mainland Chinese students in Hong Kong, not abroad (*N* = 1); (5) repeated (*N* = 1). The Chinese-language library thus contains 74 peer-reviewed articles.

Due to word count limitations, not all articles reviewed are cited in this article. However, a list of all the articles reviewed is available in Appendix 1 in Supplementary materials. In addition, it was not possible to detail the main findings of each article reviewed (Campbell & Neff, 2020).

### Analysis

Once relevant literature was identified, I analysed each of the 128 English-language and 74 Chinese-language articles (see Appendix 1 in Supplementary materials), noting (a) number of authors, (b) each author’s ethnic background (Chinese or non-Chinese), whether they received research training outside of mainland China during their higher education stages. Information of fields a-b was entered to an Excel spreadsheet and turned into a streamlined table, Table 1.

**Table 1:** Authorship comparison

	Chinese-language	English-language
Entries	74	128
Number of authors	106	211
Authors who were trained outside mainland China	19	204 (7 trained in China)
Authors who were based outside mainland China	7	173 (38 based in China)
Authors who were not ethnic Chinese	2	104 (107 were ethnic Chinese)

When the first round of data entry was completed, I conducted an inductive thematic analysis (Creswell, 2007) to determine the subject positions portrayed within the literature. I adopted a two-step approach towards thematic analysis. Firstly, I went through the abstracts and full texts of all articles to examine the key arguments made about Chinese international students; based on these arguments, I derived potential subject positions presented of the Chinese international students by drawing on the aforementioned post-colonial lens; I then placed the articles under different subject position categories. During this stage, some articles were placed under more than one subject position category. Secondly, I conducted a deeper layer of analysis to consider each subject position category (e.g. all articles that were assigned under the ‘political subjects’ category). This included reading some articles multiple times to reconsider initial categorisation and identifying alignment or contradictions with other papers under the same category. As a result, some articles were removed from initial categories, while others were added. I also eliminated or combined categories as a result of this layer of analysis and consideration. Eventually, I identified four subject positions^5^: the (1) neoliberal, (2) political, (3) pedagogic and (4) racialised subjects. The entries in the Excel spreadsheet were re-organised to be under the different categories of subject positions (Table 2). My summary and critique of each category is presented later in this article.

**Table 2:** Subject positions of Chinese international students portrayed in the reviewed literature

Subject position	Sub-categories
1. Neoliberal (*N* = 48: *N* = 12 Chinese-language + *N* = 36 English-language)	A. Cash cows B. Embedded in global neoliberal machinery, contributing to reproducing global inequalities, but also as victims
	C. Individualised without individuality vis-a-vis the Chinese state; responsibilised for own successes or failures, instrumentalised as means for achieving China’s modernisation; enterprising, calculating, striving
	D. ‘Side effect’: suffering from mental health issues
2. Political (*N* = 52: *N* = 39 Chinese-language + *N* = 13 English-language)	A. Instruments of states (both Chinese and foreign) A1: Strategic human capital for China’s modernisation A2: Cultural ambassadors or people-to-people diplomats A3: Potential spies and political/diplomatic sacrifices
	B. Ethno-national
	C. Ideological C1: Ideological mediators C2: Negotiating what democracy means and how it works C3: Malleable but also suspicious
3. Pedagogic (*N* = 38: *N* = 4 Chinese-language; *N* = 34 English-language)	A. Deficient, incompetent, reticent, lacking critical thinking
	B. Knowledgeable educators
	C. Agentic, methodical, resourceful, innovative
4. Racialised (*N* = 25: *N* = 6 Chinese-language + *N* = 19 English-language)	A. Racialised A1: Discrimination considered as racism, no further discussion A2: Postcolonial critiques & in-depth depiction of racialised experiences
	B. Ethnically diverse within China

### Researcher positionality

I was a Chinese international student for five years and have since worked as an academic publishing predominantly in English language, based in a Western university. I have had a long-standing interest in research on Chinese international students, having worked in the field for nearly a decade. When examining these two bodies of literature, I constantly interrogate myself as to whether I am making assumptions about ideological stances of literature published in either English or in Chinese and of the authors, be they Chinese or non-Chinese authors.

When I critique the Chinese-language literature, I do not assume that they are purely written with China’s indigenous framework or terms of reference; instead, I acknowledge that they may be variously influenced by Western thoughts and frameworks. Similarly, when I examine English-language publications, I also note that some scholars may have this ambition to uphold their own Chinese or original cultural tradition and inevitably are influenced by their original cultural upbringings (Yang, 2018). As such, this paper is not claiming a dichotomous distinction between the English-language and the Chinese-language literature; rather, it strives to note the shared and divergent trends and attempts to critique both through a shared set of theoretical framework—admittedly, this critique itself is biased and inevitably influenced by my own upbringing and academic training. I concur with Yang (2018, p. 41) that ‘politics of representation and authenticity’ is at the core. In this paper I strive to engage productively with ‘multiple vantage points’ to conduct more transparent and rigorous analysis. I argue that examining both the English-language and Chinese-language literature on Chinese international students is a first step forward towards this goal.

### Methodological limitations

Firstly, while this study is among the first to conduct a systematic review of both English-language and Chinese-language literature on Chinese international students, it has not included literature published in other languages. For example, during literature selection, I had to exclude 3 articles published in Korean. Future research could compare, and critique literature published in languages in addition to English and Chinese to broaden the perspectives and deepen analysis.

Secondly, this article only selected peer-reviewed journal articles, with the rationale that such publications are often viewed as possessing quality and rigour and represent the most cutting-edge work of the field (Campbell & Neff, 2020). However, there is a substantial body of literature on Chinese international students encapsulated in doctoral and master’s theses (*N* = 142) and book chapters (*N* = 12). Such works also provide fascinating insights and can potentially broaden the scope of analysis. It is therefore an avenue of future research.

Thirdly, while this article aims at having a comprehensive coverage of literature, it cannot claim to be exhaustive. This can be limited to the databases and key word combinations that I used, and more practically, the finite amount of time and resources that were available. Therefore, this literature review is necessarily limited in its coverage and this might have influenced the findings of this analysis. I recognise these limitations and hope that this article can serve as a catalyst for future scholarship to conduct even more comprehensive reviews of literature on Chinese international students.

### Presentation style

In this paper, when I cite works from the Chinese-language literature reviewed, I will provide the Chinese character of the surname of the lead author. For instance, this is how I cite Yang [杨] et al.’s (2016) work and this is how I cite Cang [苍] & Zheng’s (2016) work. Readers can thus tell the Chinese-language literature apart through the Chinese characters used. In the reference list and Appendix 1 in Supplementary materials, I include both the original Chinese information and the English translation of all Chinese-language articles reviewed. In what follows I will first present the four subject positions portrayed of the Chinese international students; I will then discuss the authorship information of the articles reviewed and offer a critique of both bodies of literature.

## Subject positions

This paper identifies four major subject positions, as shown in Table 2 above. For a detailed list of reviewed articles that fall under each subject position, refer to Appendix 2.

### The neoliberal subject

As shown in Table 2, 48 (12 Chinese-language and 36 English-language, see Table 2 and Appendix 2 in Supplementary materials) articles have portrayed aspects of the Chinese international students’ neoliberal subject position, which must be understood in two inter-related ways. Firstly, the Chinese international students are situated in foreign universities (mostly western) where neoliberalism has taken a strong hold as manifest in reduced public spending in higher education and the use of international student recruitment to make up shortfalls within university finances (Zhang & Beck, 2017); they are often ‘valued’ for their potential to contribute economically to the destination countries’ higher education and other relevant sectors, through tuition fees, and purchase of related services such as accommodation and tourism (Brooks, 2017; Yang, 2020). The literature reviewed in this paper has been proved no exception, with Chinese international students’ contribution of tuition fees highlighted in Australia (Kuang [旷] & Qi, 2016; McCrohon & Nyland, 2018), Korea (Lee, 2017), Britain (Zhang [张] 2019) and the USA (Linghu [令狐] 2019). The Chinese international students are, therefore, inevitably engulfed into the neoliberal machinery of global inequality reproduction through their fee-paying to the higher-ranked global universities and use of symbolic currency bestowed by these foreign degrees to increase their competitiveness in labour markets in China and abroad (Xu & Montgomery, 2019). Meanwhile, the Chinese international students’ exponential contribution to the global neoliberalised universities’ coffer (Chen & Ross, 2015) has often been drawn upon to justify the conduct of research into other aspects of their lives. For instance, Liu (2016, p. 3) writes:

it is likely that Chinese enrollment in American universities may not continue to increase. As the first generation of American-educated Chinese international students return to China and are not as successful as they had anticipated, there will likely be a plateau of Chinese international students applying to the United States in the near future…This will have financial implications for American universities as the approximately $33 billion in tuition and accommodation currently spent by Chinese students attending America universities decreases.

Having established the economic and cultural contributions of the Chinese international students, Liu then went on to argue for the need to listen to their voices and to ‘trace the roots’ (ibid., p. 15) of the challenges experienced by them. Such discursive work rendered by the researchers (e.g. Kuang [旷] & Qi, 2016; Liu & Vogel, 2016; Louie & Qin, 2019; Montalbano et al., 2016; Sun et al., 2020; Tao [陶] & Liu, 2016) could unwittingly create a false impression that the Chinese international students are only valuable because of their economic and instrumental contributions. This resonates with Castiello-Gutiérrez and Li’s (2020, p. 2) commentary on international students’ experiences in the US:

those who were fighting with and for us sometimes used language that unconsciously embraced a nationalistic perspective; …Such language is often taken for granted, but for us, it can be hurtful. Although without harmful intentions, these arguments perpetuate the narrative that we are commodities…and omit the fact that every single student is a story, every single one of us has a life in this country, and that our lives are inherently connected with the life of others.

As such, the Chinese international students can arguably fall victims to diverse practices of the global neoliberal universities, not only through these institutions’ ruthless revenue generation tactics and eclipse of educational missions towards international students (Su & Harrison, 2016) but also through the unwitting subscription to neoliberal values by allies such as researchers of Chinese international students. These students’ victimised position becomes more pertinent when research discussed their interactions with educational agents which were often characterised by partial or false disclosure of information by the agents, leading to a low level of satisfaction among the Chinese international students (Su & Harrison, 2016, pp. 910–911) or even detrimental mental health issues and dropouts of younger Chinese international students (Ren [任], 2018).

Secondly, not only are the Chinese international students instrumentalised by global neoliberal universities, but they are also often discussed as instruments of nation-states, including the Chinese and foreign states. They are considered as strategic human capital and talent wealth to propel the modernisation of China (Cui [崔], 2018; Jin [金], 2015; Jing [荆], 2015; Lin [林], 2017a; Linghu [令狐], 2019; Liu [柳], 2016; Wang [王] & Ding, 2020; Wu [吴] & Huang, 2017; Wu & Tarc, 2019). In Sino-Soviet relations (1950s/60s), Hu [胡] (2018) highlighted that the Chinese international students were considered as containers of knowledge to transfer advanced scientific skills back to China. Positioned as instruments of the state, Chinese international students who were not interested in state-sanctioned, prioritised subjects by both Chinese and Soviet governments were denied opportunities to study in the Soviet Union.

Relatedly, they have been subject to arguably exploitative practices by successive Chinese governments. The responsibilisation of the studying abroad project by the Chinese state is well-noted in both bodies of literature reviewed (Kajanus, 2015). Tao [陶] and Liu (2016), for instance, argued that the Chinese international students are often the sole agents to shoulder study-abroad risks and suggested the Chinese international students should learn to transfer and avoid risks through legal means. Xiao [肖] (2015) discussed how Chinese international students majoring in fine arts often cannot get their desirable jobs after returning to China and are excluded by various government talent schemes. This was due to China’s policy-making that portrayed the Chinese international students in a hierarchised manner, with certain subjects (e.g. STEM and military subjects) receiving greater policy inclinations than others.

Additionally, the Chinese international students are also depicted as rational, calculating subjects (Yan, 2010) when making decisions about where, when and how to return home or stay abroad (Tu [屠] & He, 2020; Xu, 2021) and underlining their transnational distinctions by strategically selecting reference groups, e.g. peers who were less mobile (Zhang & Xu, 2020). Frequently, the Chinese international students are found to be strategic and creative in navigating undesirable social-cultural situations in their destination countries. Yu and Moskal (2019a, 2019b) noted how the Chinese international students from a UK university’s Business School strategically engaged in Christian Church activities to circumvent the over-concentration of co-nationals in their departments. Jang and Choi (2020) showed how Chinese international students in Korea supported each other using an online community to mitigate the lack of information and chaos during the COVID-19 pandemic. Relatedly, Hu et al. (2020) demonstrated how Chinese international students drew on their families’ economic, cultural and social capital to ‘broker[] information, mobile[se] resources, and coordinat[e] disjointed acts’ (p. 1) of national and transnational institutions as disrupted by the COVID-19 pandemic. Indeed, as strategic and calculating neoliberal subjects, the Chinese international students have been portrayed as methodical in utilising their international education experience to leverage their competitive edge. For instance, Louie and Qin (2019) showed how Chinese international students used auto-owning in disindustralised Michigan to foster desired social status and ensure sociability in their projected future of returning to work and live in China. Similar accounts about how these students deployed their transnational mobilities and lives as channels of capital accumulation and upward social mobility can be found in the works of Lee (2017), Li and Pitkänen (2018), Coates (2019), Tu and Nehring (2019), Wu [吴] and Huang (2017), Zhai [翟] and Gao (2018), among others.

### The political subject

The Chinese international students’ political subject position has been revealed in nuanced ways within the two bodies of literature reviewed (*N* = 52: 39 Chinese-language and 13 English-language articles, see Table 2 and Appendix 2 in Supplementary materials). Specifically, the Chinese international students are portrayed as: (1) instruments of states—both Chinese and foreign; (2) patriotic and ethno-national subjects; and (3) ideological subjects.

Firstly, as instruments of states, the Chinese international students are notably portrayed as people-to-people diplomats to foreign countries, tasked to foster positive images of China (Bislev, 2017; Lin [林] 2016); Equally, the Chinese international students are discussed as missioned to represent interests of foreign governments when returning from the USA (Chen [陈] (2015); Yuan [元] & Yue, 2015) and the Soviet Union (Zhang [张], 2018). Interestingly, Hao [郝] (2016) and Xu [徐] (2020) detailed how the Chinese international students became targets of international competition, e.g. between the US and Japan, as both countries sought to exert political influence over China through them. Zhang [张] (2019) analysed news articles from the Guardian and suggested that such articles have focussed on reporting about Chinese international students’ economic contribution and less than desirable academic performance as a way to shift the British public attention away from the conservative government’s decreased investment in HE. The Chinese international students are, therefore, convenient political pawns for the UK government to reduce its domestic political tension.

When diplomatic relations worsen, the Chinese international students are often caught between a tug of diplomatic war between the Chinese and foreign governments. They are rendered as potential spies by the Chinese government and vice versa (Li [李], 2016), thus receiving hostile treatments from both sides. For example, the Chinese international students who studied in the Soviet Union and Japan were found to be marginalised and politically suppressed when they returned to China between 1940 and 1960s (Gan [干] & Liu, 2016; Hu [胡], 2018; Ren [任] & Liang, 2020); some were detained as suspected spies by the US government in the 1950s (Zuo [左], 2016). Such historic cases (Wang [王] & Zhang, 2018) find strong parallels in contemporary times when the Chinese international students are noted to bear the brunt of souring international relations between China and western countries. They are often used as ‘a bargaining chip to achieve diplomatic goals’ (Lau, 2020, para 5), to retaliate and censor foreign ‘interference’ with China’s contentious domestic political problems (Tran, 2020), while simultaneously being rendered as potential spies for the Chinese government, and thus are ‘security threats’ to foreign governments (Song, 2019, p. 605). As such, it appears that the Chinese international students have been, not only historically (e. g. in the 1940s/60s), but also to date, been rendered by home and receiving national governments as politically suspicious.

Secondly, the Chinese international students are often discussed as ethno-national subjects. Hail (2015, p. 1) wrote about patriotic Chinese international students concerned about their American peers’ ‘misinformed, prejudiced, offensive’ views of China and sometimes experienced ‘intense hostility’ when communicating with their American counterparts (2). Paralleled cases can be found among Chinese students in Japan (Lai, 2015). Historically, the Chinese international students have been found to be political leaders and activists both when abroad in Japan and the US (Lin [林], 2017b; Gan [干] & Liu, 2016) and when returned to China (Wang [王], 2020) in late 1800s and early 1900s.

Thirdly, the Chinese international students are also construed as ideological subjects. They were shown to be key ideological mediators in the early communication of ‘Japanese Marxism’ in China (Hu [胡], 2015). Li [李] and Sun (2019) pinpointed that contemporary Chinese international students’ negative views on China’s democracy could affect high-end talents’ motivation to return to China, hence there is a need to rectify their views by appealing to their patriotism and cultural identification. The Chinese international students’ ideological malleability is also noted by Chen [陈] (2019) who considered it important to influence the Chinese international students’ values towards ‘desirable’ directions. However, this also means that the Chinese international students can become suspicious ideological subjects. For example, in the 1950s/60s, the Chinese government carried out painstaking political background vetting of the Chinese international students before they were sent to the Soviet Union (Hu [胡], 2018; Li [李], 2016).

The Chinese international students have also been depicted as ideological subjects negotiating what democracy means and how democracy works in western contexts such as Canada (Li, 2016) and New Zealand (Zhang, 2018). They were found to be open to receiving the concepts of citizenship and democracy. However, their difficulties in getting to grips with such concepts, given the way they were brought up in China, were not always fully appreciated by Western educators. Such complexities and struggles experienced by these Chinese international students are needed in the literature.

Within this subject position, the Chinese international students have been largely instrumentalised, no matter as instruments of the state, malleable ideological subjects, or patriotic and ethno-national subjects. Always tasked with one mission or another, some Chinese international students were treated as sacrifices and pawns, without much say of their own. Exposing these problematic aspects of their political subject positions is to challenge such political suppression of the Chinese international students, and embark on ‘re-positioning’ (Xiang, 2017, p. 4) the Chinese international students onto a comprehensive and complex map of political subjectification. It should be noted, however, that this seeming lack of agency for the Chinese international students is merely an effect of the portrayal within this political subject position. We should not lose sight of these students’ personal instrumental goals of pursuing overseas education (as shown in the previous neoliberal subject position) and their epistemic contributions and agency, to be demonstrated in their pedagogic subject position.

### The pedagogic subject

In previous reviews (Henze & Zhu, 2012; Zhang-Wu, 2018), it is noted that research has placed the Chinese international students in a default ‘deficit’ position, rendering them as in need of pedagogical interventions in order to improve their inadequate language skills, insufficient contribution in class, and lack of critical thinking capability. In both bodies of literature reviewed (N = 38: 4 Chinese-language and 34 English-language, see Table 2 and Appendix 2 in supplementary materials), a small number of studies continue to portray the Chinese international students as a homogeneous, deficient pedagogic group (e.g. Liu, 2016; Liu & Vogel, 2016). The Chinese international students are suggested to be experiencing a ‘vicious circle’ that prevented them from integrating with the local society (Ma, 2017, 2020). Chinese international students studying in non-Anglophone countries are found to experience multiple-language barriers in grasping the local language(s) as well as English, e.g. in Korea (Qi [齐], 2017), Finland (Li & Pitkänen, 2018) and various European countries (Cao & Meng, 2019; Meng et al., 2018). In all, little attention was given to the Chinese international students’ social characteristics other than their English language/academic ability, e.g. their socio-economic status (Zhai [翟] & Gao, 2018), gender (Martin, 2017; Meng [孟] & Abduweli, 2017; Tu & Xie, 2020), or family backgrounds (Wu & Tarc, 2019; Wu, 2020a, 2020b, 2020c). Instead, these Chinese international students were treated as universal pedagogic subjects to be improved on.

However, diverging from this deficit approach, a nascent group of scholars have contested extant scholarship’s tendency to categorise critical thinking as a uniquely western construct (Lu [陆] & Singh, 2017). Instead, they maintained that it is more a matter of the Chinese international students not knowing the terms and conventions about how to express their critical thinking, due to lack of language competence and Western socio-cultural knowledge (Wu, 2020a, 2020b, 2020c). Concomitantly, while some Chinese international students may internalise such stereotypes and became less confident, some use their silence as ‘right, choice, resistance and strategy’ (Phan & Li, 2014, p. 233) in face of dominant educational discourses (Cai [蔡] & Welch, 2019; Ding, 2016). Wu (2020a, 2020b, 2020c, p. 2) also suggested that the stereotypes of the Chinese international students as ‘robotic learners’ may serve to evade pedagogic and moral responsibilities of western universities in providing tailor-made supports and justify their ‘indifference’ to these Chinese international students’ needs.

Indeed, some of the articles reviewed have begun to challenge normalisation of western-centric pedagogic practices and beliefs and foreground broader systemic inequalities. Chen and Ross (2015), for example, argued that the Chinese international students’ organisations on American campuses are organic networks that serve positive functions for the community. This challenges the deficit view that the Chinese international students are not integrating on campus (thus ‘threatening’ the campus diversity) and problematises the normalisation of a specific type of campus interaction. This is amply echoed in the works of Lee (2018), Chen (2019), Heng (2017, 2019), Zhao [赵] and Li (2019), and Zhang and Zhan (2020). These studies variously interrogated wider, systemic, institutionalised issues of marginalisation that led to these Chinese international students’ ‘failures’, either in academic studies or in integration. Instead, these authors advocated for demanding Western institutions to be interested in and cognisant of the Chinese international students’ backgrounds, contributions and needs.

Along the same line, Chinese international students have been portrayed as open-minded scholars and knowledgeable educators, adept at acquiring and integrating foreign and Chinese cultures. This is especially the case for Chinese international students as scholars who are highly critical and agentic, adept at intercultural learning (Ai, 2015; Dai & Hardy, 2020; Hu [胡], 2016; Hu et al., 2016; Meng [孟] & Abduweli, 2017; Xu et al., 2020; Weng, 2020) and are resourceful, strategic and tenacious (Cao & Meng, 2020; De Costa et al., 2016; Fox, 2016; Jensen, 2015; Yu & Moskal, 2019a, 2019b), showing considerable growths through their transnational education mobility; they are also celebrated for integrating Chinese thoughts with western analytical methods (Chen [陈], 2018; Lin [林], 2016; Xu [徐], 2020; Yuan [元] & Zhang, 2016).

Despite a small number of articles that continues to hold deficit presumptions about the Chinese international students, there is an increasing push back to not only challenge such deficit views, but also to reverse the discourse to evidence that the Chinese international students are epistemic equals of their western counterparts (Walker, 2019), capable of making significant intellectual contribution to the humankind. This nascent body of literature interrogates power hierarchies and the normalisation of western-centric pedagogic assumptions and prescriptions. It has advocated to consider the Chinese international students’ pedagogic needs as legitimate and necessary, without compromising their subjectivity as ‘problematic’ or ‘in need of fixing’. Importantly, such literature has asked for western institutions to reconsider their relations with the Chinese international students and establish an equal partnership to recognise the fact that they have much to learn from the Chinese international students, thus contributing to recognising the epistemic contributions of the Chinese international students.

### The racialised subject

Chinese international students have inhabited marginal racial spaces in Western contexts when studying overseas. Research has shown that they are either positioned as part of the ‘model minority’, lumped together with American Chinese or British Born Chinese, or their racial identity is under-played (Zhang-Wu, 2018). Compared with the race-blind and colour-blind tendency displayed in the USA-focussed studies reviewed by Zhang-Wu (2018), there is a notable group of articles (*N* = 25: 6 Chinese-language and 19 English-language, see Table 2 and Appendix 2 in Supplementary materials) that engages with the Chinese international students’ racialised subject position. There are, however, different characteristics shown.

Firstly, a group of articles touched upon the Chinese international students’ experienced and perceived discriminations but did not probe further into the structural issues or explore wider impacts. For instance, Zhang and Jung’s (2017, p. 35) study on acculturative stress experienced by Chinese international students in the US found the Chinese international students were ‘likely to attribute the feeling of being rejected to discrimination’, but did not go further than suggesting a need for further research. In Meng et al. (2019) research that explored Chinese international students’ intercultural interaction with cultural ‘others’ in Belgium, they stopped at suggesting the importance to foster ‘a friendly and receptive campus and local community’ (ibid.). Similar approaches can also be detected in the works of Lu et al. (2018), Lin [林] and Chen (2019), and Zhu and Bresnahan (2018a; 2018b) and in historic accounts of the Chinese international students (Cang [苍] & Zheng, 2016; Hao [郝], 2016; Zhang [张], 2016).

In contrast, a second group conducted critical analysis of structural issues, e.g. Suspitsyna and Shalka’s (2019) aforementioned post-colonial analysis of how Chinese international students were portrayed in media representation within Chronicle of Higher Education and Zhang-Wu’s (2018) review of research about Chinese international students in America. Both papers offered critiques of the problematic, colonial nature of media and academic representations of the Chinese international students in the American context. Additionally, an emergent group of empirical work directly engages with the Chinese international students’ racialised experiences, either as victims of neo-racism and the ‘other’ in American colleges (Yao, 2018), or as informed agents of naming practices demonstrating acute racial and ethnic awareness of what names meant for their ethnic identities (Fang & Fine, 2020).

The Chinese international students are also found to have fluid racial identifications, either being internally conflicted about their identities as Chinese and as being ‘Americanised’ (Valdez, 2015) or as their lengths of stay abroad increased, shifting from identifying as Chinese to being ‘Asian’ (Okura, 2019). This new attention to how the Chinese international students ‘learn about, encounter, and navigate “race”’ (ibid., 1) is further developed by Martin (2020) who exposed the simplistic ways that some Chinese international students in Melbourne categorised different racial groups in their naïve engagement with racialised discourses and inter-racial relations. These studies thus depart from the Chinese international students being passive recipients of racialised treatments but accentuate their roles as discerning racialised subjects.

While there is an emergent effort among scholars to research the racialised experiences of the Chinese international students, much more in-depth and theoretical work is necessary. Research on this subject position directly engages with issues of colour-blind tendency, post-colonial subjugation, othering and neo-racism, which are all pivotal theoretical and conceptual tools for this subfield. There is, however, a dire lack of research attention devoted to acknowledging the ethnic diversity among the Chinese international students, with only one English-language article (Sude et al., 2020) and no Chinese-language publication to address this aspect.

## Discussion and implications

Regarding the authorship of these reviewed articles, it can be observed in Table 1 that around half of the authors for the English-language articles are ethnic Chinese, while for the Chinese-language literature, only 2 out of 106 authors are not of ethnic Chinese backgrounds, and both were second authors. 38 out of 211 English-language articles’ authors are based in China. In contrast, only 7 out of 106 authors of the Chinese-language literature were based outside of mainland China. These figures suggest that the Chinese-language articles are overwhelmingly published by authors who were ethnic Chinese and were based in mainland China at the time of publication. It should be noted that this article makes no attempt to establish a direct relationship between the authors’ ethnic background or places where they received research training, and their research or ideological orientations in their work on Chinese international students. Rather, this article provides this set of information to help readers contextualise the specifics of knowledge production, i.e. of research on Chinese international students in this case. This is particularly relevant to (1) the discussion below about potential reasons for the divergence of research focuses and approaches between the English-language and Chinese-language literature; and (2) the inequalities of knowledge production between the global West and East (see section on the ‘post-colonial lens), a divide within which research literature on Chinese international students is situated (Yang, 2018). Indeed, the authorship information has implications on the academic and political environment in which these authors operated within.

The Chinese-language literature has a substantial focus on historical studies of Chinese international students, especially on the modern China period (i.e. Qing Dynasty and Republic of China era). This is unparalleled in the English-language literature, which is predominantly concerned with the contemporary. Such historical insights have highlighted continuities and changes about the Chinese international students’ subject positions, for instance, their intellectual power and epistemic contributions to knowledge as pedagogic subjects. This emphasis contrasts with some of the English-language literature’s still present focus on the Chinese international students as deficient pedagogic subjects who needs ‘improvement’ through interventions. Intriguingly, the Chinese-language literature has also displayed a predominant attention to the Chinese international students’ political subject positions, e.g. their position as political and diplomatic pawns and sacrifices, rampant in WWI and WWII periods. Unsurprisingly, during such turmoil, the unstable economic and political situations were such that Chinese international student returnees encountered a great deal of difficulties in securing employments. Chinese international students of certain disciplinary backgrounds were treated unfairly by successive Chinese governments because they could not ‘fulfil’ the state’s intended ‘functions’. These are all continuities that resonate with the experiences of today’s Chinese international students. The Chinese-language literature thus establishes a platform for historicising the political and epistemic experiences of the contemporary Chinese international students.

In contrast, the Chinese-language literature that focuses on contemporary Chinese international students has largely shunned their political subject position. This could be to do with the censorship and self-censoring that are rampant within mainland China. According to Guiheux and Wang (2018, p. 25), much research in China is state funded or sponsored by local governments. This funding mechanism predisposes that publications are ‘constrained by the current political line’, making it impractical and even risky to be critical of contemporary public policies (Chou, 2014). Comparatively, conducting historic research and being critical of previous Chinese governments’ policies may not be as risky.

However, censorship and self-censoring alone cannot adequately explain such features of the Chinese-language literature. For instance, this review has found that the Chinese-language literature is also deeply concerned about the Chinese international students as ideological subjects whose value systems and patriotism can have implications for the future of China. Such literature is therefore keen to propose ways to ‘mould’ the Chinese international students’ ideological beliefs. This can be informed by the notion of the scholar/researcher in Chinese society, who is said to ‘have a larger responsibility and more positive role in society and in relation to the state’ (Marginson, 2014, p. 35). Such responsibility ‘includes responsibility for the good order and stable reproduction of state and society’ (ibid.). The Chinese scholar is thus tasked with not only producing knowledge, but also using the power bestowed on them wisely in the ‘collective interest’ (ibid., p. 36). Judging from the focuses of many of the Chinese-language articles, the harmony and stability of the society seem to trump all other ‘collective interest’. The Chinese-language literature thus displays a notable tendency to use research to serve the ruling elites’ political interests.

In comparison, the English-language literature pays relatively less attention to the Chinese international students’ political subject positions, when compared with the pedagogic, neoliberal and racialised positions. This suggests a lingering dominance of the colonial power within western higher education. The Chinese international students are still primarily valued for and warranted research attention due to their subjectification as willing contributors to pumping up the dwindling HE funds in the west. Their neoliberal subjectivities as globally mobile, enterprising individuals fit seamlessly with the neoliberalised western universities to reproduce and exacerbate global inequalities (Ross & Chen, 2015; Yang, 2020). It appears that the English-language literature is grappling with proving that the Chinese international students are not inferior epistemic subjects, whose mimicry (e.g. reception of western instructions) is never sufficient (Suspitsyna & Shalka, 2019). The Chinese international students continue to be ‘wronged specifically in [their] capacity as a knower; [they] do not have a voice that is recognised’ (Walker, 2019, p. 165), which is defined as a form of ‘epistemic injustice’. Such epistemic injustice, according to Song (2019, p. 608), stems from the Occident’s sense of superiority that relegates ‘other peoples as having a lower degree of intellectual and moral development and different forms of rationality as inferior’. It is therefore heartening to see a nascent body of literature from both languages (see ‘the pedagogic subject’ section) that challenges the universal superiority of ‘westernised’ critical thinking, accentuates the epistemic qualities and intellectual contributions, wide-ranging agencies, and legitimate pedagogic needs of the Chinese international students.

In both bodies of literature reviewed, the various nation-states’ instrumentalisation of the Chinese international students as neoliberal and political subjects have revealed problematic treatments rendered upon the Chinese international students. It is fair to suggest that in these respects, nation-states (both Chinese and foreign), HEIs, the media and even certain researchers have, to various degrees, exploited the vulnerabilities of the Chinese international students. This recognition thus goes beyond existing post-colonial critiques that have been proffered by seminal works of scholars such as Suspitsyna and Shalka (2019) and Moosavi (2021). It points out the joint oppression (Freire, 2017; Jebril, 2021) inflicted on the Chinese international students by a host of powerful actors.

This critique, however, acknowledges that the Chinese international students too, play a part in producing and exacerbating global inequalities through their acquiescence in global higher education hierarchies and paying of exorbitant tuition fees (Yang, 2020). Some Chinese international students may have also internalised the pathologisation of their alleged lack of critical thinking ability. Therefore, another important research and educational direction for our community is to raise the consciousness of the Chinese international students about this host of inequalities revolving around their subject positions and pinpoint their own roles.

Moreover, different from the monolithic representation of Chinese international students in most existing research, policy and media, it is heartening to witness an emergent body of literature that reveals the heterogeneity of these Chinese students. For instance, while a dominant body of research in both languages still focuses on undergraduate and master’s level Chinese international students’ experiences, a nascent group has begun to probe into various aspects of the lives of pre-university (Montalbano et al., 2016; Ren [任], 2018; Will, 2017; Wu & Tarc, 2019; Wu, 2020a, 2020b), vocational education (Cao & Tran, 2015) and doctoral level students (Ding, 2016; Ding & Devine, 2017; Hu et al., 2016; Weng, 2020; Xu et al., 2020; Yue [岳], 2015). There has also been research that accentuates their diverse gender and sexual experiences (Douglass et al., 2020; Martin, 2017), emotional lives (Coates, 2019; Montsion, 2020) and religious beliefs (Chen [2015; Yu, 2019). Due to space constraint, this article cannot get into more details of these aspects, but they together demonstrate the notable heterogeneity of the Chinese international students.

## Future directions and conclusion

This paper has pointed to three future research directions. Firstly, considering the strengths of the Chinese-language literature, greater communication of research agendas and approaches between the English- and Chinese-language research communities seems beneficial. This is not only relevant to research on Chinese international students, but can be applied to broader research that concerns internationalisation of Chinese education (Yang, 2018). Secondly, it is pivotal for research to continue accentuating and legitimating the epistemic qualities, agencies, and contributions of Chinese international students while highlighting the corresponding epistemic injustice exercised on them by governments and institutions. Thirdly, research and education should aim to raise the Chinese international students’ consciousness of their own roles in reproducing global higher education inequalities and in internalising pernicious aspects of their portrayed subject positions. Scholars publishing in both English and in Chinese about Chinese international students and beyond can, therefore, capitalise on the wealth of epistemic traditions from both China’s indigenous and Western perspectives, to enrich our theoretical, conceptual and empirical understandings towards the Chinese international students as a phenomenon and a constituent of contemporary global higher education.

By reviewing and critiquing 128 English-language and 74 Chinese-language articles on Chinese international students published between 2015 and 2020, this article makes important interventions to not only research on Chinese international students, but also knowledge production in the context of global inequalities. In making contribution to research on Chinese international students, this article points out that while the English-language literature still grapples with ‘proving’ Chinese international students as epistemic equals of their Western counterparts, thus imposing ‘epistemic injustice’ on these students, the Chinese-language literature celebrates the intellectual, social and cultural contributions made by successive generations of Chinese students/scholars, both at home and abroad. This has been achieved through the latter’s substantial focus on historical studies of these students, thus demonstrating strengths of historicising research on international students. However, when approaching the political subject positions of these Chinese international students, the latter’s predominant historical focus and apparent lack of contemporary discussion may be related to issues of censorship and ‘scholarly responsibility’ placed on shoulders of researchers, as conditioned by the unique socio-political context of China today (Guiheux & Simeng, 2018; Marginson, 2014).

In making contributions to global knowledge production, this article is among the first to bring forth a dialogue between the dominant English-language and the indigenous Chinese-language literature. This act challenges the hierarchies of knowledge production, through accentuating the strengths, legitimacy and contributions of the Chinese-language literature. The fruitful comparative insights yielded thus helps to foreground the problematic subject positions portrayed of the Chinese international students, serving as salient evidence to question the normalisation of western-centric assumptions and practices in international education provision. This evidence also sets in motion our quest to placing Chinese international students and indeed Chinese-language scholarships on these students as epistemic equals of their western counterparts.

## Supplementary Information

Below is the link to the electronic supplementary material.

Supplementary file1 (DOCX 51 kb)

## Footnotes

1 Within this article, Chinese international students refer to higher education and high school students who move from the Qing Dynasty (1636–1912), Republic of China (1912–1949) and People’s Republic of China (1949-present), including Hong Kong and Macau, to study in other countries. The term also encompasses Chinese international students who have returned to the country after their sojourns, commonly referred to as returnees in the literature.

2 Note that the * symbol used in these searching exercises was for variable endings of the searching terms, e.g. the singular and plural forms of ‘Chinese international/overseas student(s)’.

3 Those that were excluded only mentioned ‘Chinese international students’ in passing without having them as focuses of research.

4 Chinese Social Sciences Citation Index (CSSCI) is an interdisciplinary citation programme in China. Established in 2000, this citation database covers about 500 Chinese academic journals of humanities and social sciences. Now many leading Chinese universities use CSSCI as a basis for the evaluation of academic achievements and promotion.

5 Initially 8 subject positions were identified, including (1) economic, (2) neoliberal, (3) political, (4) pedagogic, (5) caring/feeling, (6) racialised/ethnic, (7) gendered/sexual, (8) religious subjects. However, due to space constraints, this article has chosen to focus on four of the most substantial subject positions. It is anticipated that another article will be published regarding the other four subject positions.
