# Supplementary material for: Portraying the ‘Chinese international students’: a review of English-language and Chinese-language literature on Chinese international students (2015–2020)
Source: Asia Pac Educ Rev. 2021 Nov 27;23(1):151–67. doi: 10.1007/s12564-021-09731-8 (PMC8626731; doi:10.1007/s12564-021-09731-8)
Supplement: Supplementary file 1 — Supplementary file1 (DOCX 51 kb) [file 12564_2021_9731_MOESM1_ESM.docx]

**Appendixes 1-2**

**Appendix 1. All Articles Reviewed**

***Chinese-language Articles (N=74)***

1. Bu [卜], Y. 元. S. 石. (2019). Review and prospect of Chinese law students studying in Germany for 40 years - An investigation based on their doctoral theses [中国法科学生留学德国四十年的回顾与展望——基于博士学位论文的考察]. *Chinese Journal of Law [法学研究], 41*(02), 3-22.
2. Cai [蔡], L. 连. Y. 玉., & Welch, A. (2019). What do Chinese Overseas Graduate Students' Encounter in Australia?—A Narrative Inquiry into Their Academic [中国留学研究生在澳大利亚遭遇了什么:学术与文化适应的叙事研究]. *Studies in Foreign Education [外国教育研究], 46*(06), 89-104.
3. Cang [苍], E. 尔. Q. 青., & Zheng [郑], Y. 滢. X. 轩. (2016). Studying abroad at a young age leading to the lack of a sense of national cultural belonging [低龄留学导致民族文化归属感的缺失]. *Journal of Jishou University (Social Sciences) [吉首大学学报(社会科学版)], 37*(S2), 148-149.
4. Chen [陈], D. 丹. (2018). The population and relevant issues of Chinese overseas returnee students from the USA in 1950s [二十世纪五十年代归国留美学人群体及相关问题研究刍议]. *CPC History Studies [中共党史研究]*(03), 114-121.
5. Chen [陈], S. 思. (2018). Research on University Students’ Nationalistic Values under Different Institutional Environments ——Based on a Comparative Analysis between Chinese Students Studying in America and Mainland China [不同制度环境下的大学生民族主义价值观——基于对中国高校学生与在美留学生的比较研究]. *Henan Social Sciences [河南社会科学], 26*(06), 107-111.
6. Chen [陈], S. 思. (2019). Research on The Influence of Economic Institutional Environment on Economic System Values of University Students: Based on Comparative Analysis on Chinese University Students and Chinese Students in The United States [经济制度环境对大学生群体价值观的影响研究——基于中美高校在校中国学生的比较分析]. *Journal of University of Jinan (Social Sciences Edition) [济南大学学报(社会科学版)], 29*(05), 143-148+160.
7. Chen [陈], Y. 燕. (2015). Studying through Attracting but not Teaching: A Case Study on How Christian Churches in the United States Approach and Affect Chinese Students [既“来学”,则“往教”:美国教会接触和影响中国留学生的个案研究]. *Overseas Chinese History Studies [华侨华人历史研究]*(01), 56-66.
8. Chu [楚], X. 雪., & Zhang [张], G. 国. L. 良. (2019). The Influence of Internet Use on the Cultural Identity of Chinese Students in the United States [互联网使用对留美中国学生文化认同的影响]. *Journalism Research [新闻大学]*(05), 74-86+119.
9. Chu [楚], X. 雪., & Zhang [张], G. 国. L. 良. (2020). The Influence of Internet Use on the Cultural Identity of Chinese International Students - based on a comparative study of Chinese and non-Chinese students in the United States [互联网使用对中国留学生文化认同的影响—基于留美中国与他国学生的比较研究]. *Journal of Southwest Minzu University (Humanities and Social Science) [西南民族大学学报(人文社科版)], 41*(05), 164-169.
10. Cui [崔], X. 晓. L. 麟. (2018). Analysis of the Driving Causes for Policy Change Concerning Returned Overseas students in the 40 Years of Reform and Opening-Up [改革开放40年留学回国政策变迁动因分析]. *Journal of Guangxi University for Nationalities (Philosophy and Social Science Edition) [广西民族大学学报(哲学社会科学版)], 40*(06), 210-216.
11. Fu [付], X. 晓. Y. 燕. (2018). “Culture Shock” and Cultural Identity in Cyberspace: based on the Life Stories of Chinese International Students' Social Media Use [网络空间的“文化休克”与文化认同:基于中国留学生社交媒体使用的生命故事]. *Chinese Journal of Journalism & Communication [国际新闻界], 40*(03), 63-82.
12. Gan [干], B. 保. Z. 柱., & Liu [刘], X. 笑. F. 非. (2016). Early after World War Ⅱ National Identityof Chinese Students Studying in Japan [战后初期中国留日学生的国家认同]. *History Teaching [历史教学(下半月刊)]*(11), 43-51.
13. Gao [高], X. 晓. R. 瑞. (2017). Overseas study experience and 1920s literary disputes [留学体验与1920年代文学论争]. *Seeker [求索]*(03), 177-182.
14. Hao [郝], T. 天. H. 豪. (2016). A study of the phenomenon of studying abroad in multiple countries in modern China -- based on an analysis of statistical data [近代中国一人留学多国现象研究 —基于数据统计的分析]. *Modern University Education [现代大学教育]*(05), 62-70.
15. Hu [胡], F. 芳. Y. 毅. (2016). The improvement of cross-cultural adaptability of college students in the Internet age [互联网时代大学生跨文化适应能力的提升]. *China University Teaching [中国大学教学]*(09), 32-35.
16. Hu [胡], W. 为. X. 雄. (2015). Chinese students studying in Japan and the early communication of ‘Japanese Marxism' in China [赴日留学生与“日本马克思主义”在中国的早期传播]. *Marxism and Reality [马克思主义与现实]*(03), 24-31.
17. Hu [胡], X. 晓. J. 菁. (2018). Research on the Students of Chinese Academy of Science Dispatched to the Soviet Union [中国科学院选派留苏生的探索]. *The Chinese Journal for the History of Science and Technology [中国科技史杂志], 39*(04), 456-466.
18. Jiang [姜], Z. 贞., & Jiang [蒋], X. 晓. T. 涛. (2017). Modern Chinese international students and the Chinese film industry in the 1920s [近代留学生与20世纪20年代中国电影产业]. *Movie Review [电影评介]*(01), 15-24.
19. Jiang [江], G. 国. H. 华., & Han [韩], Y. 玉. T. 亭. (2017). Chinese international students in France and the modernisation of China's legal system in the late Qing Dynasty and early Republic of China periods [清末民初法科留学生与中国法制近代化]. *Seeker [求索]*(01), 199-204.
20. Jin [金], B. 兵. (2015). Returned Students and Vocational Guidance in Modern China [海外归国留学生与近代中国职业指导事业]. *Overseas Chinese History Studies [华侨华人历史研究]*(01), 67-75.
21. Jin [金], S. 闪. S. 闪., & Guo [郭], F. 凤. Y. 英. (2018). A study of cross-cultural ability and adaptability of Chinese students- Taking the master's in law and engineering Chinese students from the China-France Cooperative College as an example [中国留学生的跨文化能力与适应性研究——以中法合作学院留法工科硕士生为例]. *Modern University Education [现代大学教育]*(03), 94-102.
22. Jing [荆], Y. 月. X. 新. (2015). The Innovations and Its Deficiencies of the Returned Students Officer － Selecting System in the Late Qing Dynasty [清末留学生选官制度的机制创新及其局限]. *Chinese Public Administration [中国行政管理]*(12), 80-84.
23. Kuang [旷], Q. 群., & Qi [戚], Y. 业. G. 国. (2016). An analysis on the origins of 'overseas studies fever' to Australia - based on push-pull factor theory [赴澳“留学热”探源——基于推拉因素理论的分析]. *Higher Education Exploration [高教探索]*(01), 20-26.
24. Li [李], P. 鹏. (2016). New China's overseas education research in 1960s [20世纪60年代新中国留苏教育研究]. *Contemporary World and Socialism [当代世界与社会主义]*(02), 190-198.
25. Li [李], Q. 强., & Sun [孙], Y. 亚. M. 梅. (2019). How Chinese and foreign students comment on China's economic and social development -- An aurvey analysis of Chinese, British and Germany students' social attitudes [中外大学生如何评价中国经济社会发展——中、英、德三国大学生社会态度问卷调查分析]. *Jianghai Academic Journal [江海学刊]*(05), 117-123.
26. Li [李], Q. 清. (2019). Overseas education and Yuanpei Cai's communication of Western aesthetics [留学教育与蔡元培对西方美学的传播]. *Modern Communication (Journal of Communication University of China) [现代传播(中国传媒大学学报)], 41*(01), 158-163.
27. Li [李], X. 雪. T. 涛. (2019). On the Research Paradigm of the History of Chinese Students Studying Abroad ——Reflections Inferred from History of the Spirit of Overseas Chinese Studying in Japan [留学史研究范式的评价与反思——以《近代中国人留日精神史》为中心的讨论]. *Exploration and Free Views [探索与争鸣]*(04), 133-139+160.
28. Lin [林], W. 伟. (2016). Learn to be cosmopolite: Chinese students in the Cosmopiltan Club Movement (1903-1914) [学做世界公民:留美中国学生与世界会运动(1903-1914)]. *Journal of Higher Education [高等教育研究], 37*(03), 87-93.
29. Lin [林], W. 伟. (2017a). How China’s Peiyang University Sent Students Abroad to Study in Harvard University in 1906: A Historical Recount [1906年北洋大学资送学生留学哈佛大学考释]. *Modern University Education [现代大学教育]*(05), 56-63+113.
30. Lin [林], W. 伟. (2017b). Wild wishes and disappointments: Expectations and protests of Chinese international students in the United States before and after the Paris Peace Conference [奢望与失望:巴黎和会前后留美中国学生的期待与抗议]. *Theory Monthly [理论月刊]*(05), 77-82.
31. Lin [林], X. 煦. D. 丹., & Chen [陈], X. 晓. L. 亮. (2019). Stereotype and identity construction under the background of transnational education mobility" A case study of Chinese international students in the United States [跨国教育流动中的刻板印象与身份建构——以在美中国留学生为例]. *Human Geography [人文地理], 34*(03), 68-74.
32. Linghu [令狐], P. 萍. (2019). Chinese Students Studying in the USA in the New generation: Influence, Characteristics and Trend [新生代中国留美学潮:影响、特点及趋势]. *Journal of Shenzhen University (Humanities ＆ Social Sciences) [深圳大学学报(人文社会科学版)], 36*(01), 104-112.
33. Liu [刘], H. 红., & Yu [余], W. 文. D. 都. (2015). Investigating reasons for Chinese study-abroad students' educational translation activities after the First Sino-Japanese War [甲午战后中国留学生教育翻译活动兴起探因]. *Educational Research and Experiment 【教育研究与实验】*(06), 72-77.
34. Liu [刘], Q. 启. P. 鹏. (2015). Study-abroad students in France and the modern transformation of Chinese arts education [赴法留学生与中国美术教育的现代转型]. *Continue Education Research [继续教育研究]*(12), 114-116.
35. Liu [柳], X. 学. Z. 智. (2016). Analysis on development trends for Chinese students studying abroad and coming back [中国学生出国留学和留学回国发展趋势分析]. *Chinese Public Administration [中国行政管理]*(01), 52-57.
36. Lu [陆], S. 思. Y. 逸., & Singh, M. (2017). On the Constructions of Chinese students as Uncritical Thinkers in the West [中国留学生批判性思维的中西割裂]. *Modern University Education [现代大学教育]*(03), 41-48+113.
37. Luo [罗], S. 时. M. 铭. (2016). Fulin Xu-- A brief investigation into the first Chinese international student majoring in physical education in China's history [徐傅霖——中国历史上的第一个体育专业留学生小考]. *Sports Culture Guide [体育文化导刊]*(01), 180-182+200.
38. Lv [吕], C. 催. F. 芳. (2017). Researching psychological and social cultural adaptability of Chinese international students in the USA [中国在美留学生心理和社会文化适应质性研究]. *Education Research Monthly [教育学术月刊]*(05), 3-13.
39. Ma [马], W. 万. H. 华., & Kuang [匡], J. 建. J. 江. (2016). International mobility: Challenges faced by Chinese international students in the UK [国际流动:留英中国学生面临的挑战]. *Peking University Education Review [北京大学教育评论], 14*(02), 177-186.
40. Meng [孟], X. 霞., & Abduweli [阿不都外力], M. 美. (2017). Research on female study-abroad students' cross-cultural life adaptation--based on an empirical investigation on Chinese international students in the USA [女留学生跨文化生活适应研究——基于对中国在美国留学生的调研]. *Xinjiang Social Science [新疆社会科学]*(06), 174-178.
41. Qi [齐], Y. 艳. (2017). A Qualitative Research of College Graduates’ Path Choice for Studying Abroad and Adaptation [高校本科毕业生赴外深造之路径选择与适应的质性研究]. *Heilongjiang Ｒesearches on Higher Education [黑龙江高教研究]*(12), 125-129.
42. Ren [任], C. 慈., & Liang [梁], M. 茂. X. 信. (2020). Diplomacy and Immigration: A Study of Chinese Students in the U．S． during the Early Cold War Period (1948－1957) [从外交到移民——美国对中国“滞留”学生政策的转变分析(1948～1957)]. *The Chinese Journal of American Studies [美国研究], 34*(02), 86-103+106-107.
43. Ren [任], J. 杰. H. 慧. (2018). Non-continuous Education: A Study of the Adaptability of Young Chinese Overseas Students [非连续性教育:中国小留学生海外适应性问题研究]. *Journal of Research on Education for Ethnic Minorities [民族教育研究], 29*(01), 116-122.
44. Sun [孙], X. 晓. Y. 云. (2016). A brief discussion on the origins and development of female overseas study in late Qing dynasty [浅谈晚清女子留学的兴起与发展]. *History Research and Teaching [历史教学问题]*(04), 82-85.
45. Tan [谭], Y. 瑜. (2018). Application of Ethnographic Methods in Cross-cultural Learning and Practice of Chinese Overseas Students [民族志方法在中国留学生跨文化学习与实践中的运用]. *Journal of Research on Education for Ethnic Minorities [民族教育研究], 29*(06), 134-140.
46. Tao [陶], M. 美. Z. 重., & Liu [刘], H. 红. (2016). A Probe into the Consumption Risk of Overseas Education under the Background of Studying Abroad [留学潮背景下个人境外教育消费风险评析]. *Fudan Education Forum [复旦教育论坛], 14*(05), 102-107+112.
47. Tu [屠], M. 梦. W. 薇., & He [何], X. 雪. S. 松. (2020). From Studying Abroad to Employment: How Time Influences the Immigration Decision-making [从留学到就业:时间如何影响跨国迁移决策]. *Research of Youth [青年研究]*(03), 27-37+94-95.
48. Wang [王], A. 安. Y. 轶., & Ding [丁], Z. 兆. J. 君. (2020). The return of science and technology professionals returned to China in the early years of the People's Republic of China [新中国成立之初留学归国的科技工作者]. *Science & Technology Review [科技导报], 38*(10), 82-89.
49. Wang [王], J. 京. H. 浩., & Zhang [张], L. 藜. (2018). The Historical Process of the Return of Pao-tung Huang to the Chinese Mainland: Factor Analysis of the Motivation of Chinese Students Which Studied in the United States Ｒeturned to the Chinese Mainland in the 1950s [黄葆同归国始末:1950年代留美学生回国因素探析]. *Chemistry [化学通报], 81*(06), 571-575.
50. Wang [王], J. 静. (2016). American society's acceptance of Chinese international students in the early twentith century [20世纪初美国社会对中国留学生的接纳]. *Jiangxi Social Sciences [江西社会科学], 36*(10), 153-159.
51. Wang [王], K. 凯., Min [闵], Q. 庆. P. 鹏., He [何], J. 江. C. 川., Yang [杨], F. 放., & Qin [覃], L. 利. (2015). Comparison of psychological characteristics between Chinese students studying in Thailand and Thai college students [在泰中国留学生与泰国大学生心理结构特征分析]. *Chinese Journal of School Health [中国学校卫生], 36*(08), 1170-1172.
52. Wang [王], Z. 志. T. 通. (2020). The Salvation of Literati: Why Chen Tian-hua and Liang Ji Drowned Themselves [为国身死:陈天华与梁济的投水自杀]. *Social Science of Beijing [北京社会科学]*(02), 75-84.
53. Wei [韦], Q. 庆. Y. 媛. (2018). Research on the overseas students studying the library science in the Republic of China era [民国时期图书馆学留学生群体的构成及分析]. *Journal of Academic Library [大学图书馆学报], 36*(03), 102-118.
54. Wu [吴], J. 建. J. 军., & Huang [黄], D. 丹. (2017). A study on the effects of space diffusion by drawingon Chinese international returnee students' technological progress [中国留学生回流的技术进步空间扩散效应研究]. *Seeker [求索]*(09), 73-79.
55. Xiao [肖], L. 龙. F. 飞. (2015). A Preliminary Study of the Chinese Overseas Arts Students during the Recent Years [近年来中国艺术留学生初探]. *Overseas Chinese History Studies [华侨华人历史研究]*(04), 84-89.
56. Xiao [肖], Y. 玥. (2017). A review of the literature on foreign trade thought of Chinese overseas students in modern China [中国近代海外留学生对外贸易思想文献述评]. *Journal of Zhongnan University of Economics and Law [中南财经政法大学学报]*(03), 148-155.
57. Xu [徐], Z. 志. M. 民. (2020). Japan's research on Chinese international students in Japan in modern times [日本的近代中国留日学生研究]. *Modern Chinese History Studies [近代史研究]*(01), 147-159.
58. Yang [杨], L. 柳., Fu [傅], N. 纳., & Wang [王], M. 孟. N. 楠. (2019). Influencing factors of Chinese overseas students' socio-cultural adaptation [中国留学生社会文化适应的影响因素研究]. *Journal of Education Studies [教育学报], 15*(06), 93-101.
59. Yang [杨], M. 茂. Q. 庆., Huang [黄], R. 如., & Yan [严], W. 文. Y. 宜. (2016). Overseas students and the establishment of comparative education in the period of the Republic of China民国时期留学生群体与中国比较教育学科的创建. *Journal of Guangxi Normal University: Philosophy and Social Sciences Edition [广西师范大学学报(哲学社会科学版)], 52*(02), 154-158.
60. Yao [姚], Q. 琦. (2016). A comparative analysis of Chinese overseas education to Europe, America and Japan in modern China [中国近代赴欧美与赴日本留学教育比较分析]. *Educational Review [教育评论]*(11), 159-162.
61. Ye [叶], L. 荔. H. 辉., & Wang [王], J. 坚. (2018). Chinese Overseas Students' Acculturation Orientations and Career Adaptability: The Mediation Role of Career Exploration [中国籍留学生跨文化适应与生涯适应的关系:职业探索的中介作用]. *Studies of Psychology and Behavior [心理与行为研究], 16*(03), 408-413.
62. Yuan[元], Q. 青., & Yue [岳], T.-t. 婷. (2015). The development progress and tendency of studying abroad in America in the new period [新时期中国留美教育的发展历程和趋势]. *Contemporary China History Studies [当代中国史研究], 22*(01), 65-76+126-127.
63. Yuan[元], Q. 青., & Zhang[张], L.-y. 连. (2016). The Studies of Chinese Politics by Overseas Chinese Students during the Republican Period: A Survey Based on Their Dissertations [民国时期留美生中国政治问题研究的海外评价——以留美生博士论文为中心的考察]. *Overseas Chinese History Studies [华侨华人历史研究]*(04), 58-65.
64. Yue [岳], T. 婷. T. 婷. (2015). Research on American Educated Chinese PhDs Since the Reform and Opening-UP [改革开放以来的中国留美博士群体研究]. *Journal of Lanzhou University (Social Science) [兰州大学学报(社会科学版)], 43*(02), 157-163.
65. Zhai [翟], K. 柯. Y. 宇., & Gao [高], X. 兴. (2018). Social Mobility of Chinese PhD Graduates from Britain [中国留英博士毕业生的社会流动]. *Contemporary Youth Research [当代青年研究]*(01), 60-66.
66. Zhang [张], B. 宝. Q. 强. (2016). Cultural Traits and Historic Mission of Chinese Overseas Students Majoring in Sports [论体育专业留学生的文化特质与历史使命]. *Journal of Beijing Sport University [北京体育大学学报], 39*(06), 36-43+50.
67. Zhang [张], C. 粲. (2020). Chinese international students' translation of and research on Dream of the Red Chamber in France in 1930s [20世纪30年代旅法中国留学生对《红楼梦》的翻译与研究]. *The Journal of Ming-Qing Fiction Studies [明清小说研究]*(02), 262-276.
68. Zhang [张], H. 慧. J. 婧. (2016). From“ Job-housing Space”to“ Social Space”：The Adaptation Strategies of New Chinese Immigrants in Japan [从“职住空间”到“社会空间”——在日中国新移民的生存适应策略探讨]. *Overseas Chinese History Studies [华侨华人历史研究]*(03), 17-26.
69. Zhang [张], J. 建. H. 华. (2018). Chinese Students Studying in the Soviet Union in the 1950s and 1960s and the Image of the Soviet Union in Their Eyes [20世纪五六十年代的留苏学人及其视野中的“苏联形象”]. *Journal of Overseas Chinese History Studies [华侨华人历史研究]*(01), 52-60.
70. Zhang [张], L. 露. X. 茜. (2019). The International Image of Higher Education in China: A Study of The Discourses of the British Media (2001-2017) [中国高等教育国际化形象:英媒历时话语研究(2001-2017年)]. *Higher Education Exploration [高教探索]*(04), 30-36.
71. Zhang [张], S. 思. Q. 齐. (2018). Impacts of 'strong ties' in social networks on overseas studies - an empirical investigation on Chinese international students in the London School of Economics and Political Science [社会网络中的“强连接”对留学生活的影响——基于英国伦敦政治经济学院中国留学生的实证研究]. *Jianghan Tribune [江汉论坛]*(01), 138-144.
72. Zhang [章], H. 宏., & Hu [胡], H. 颢. C. 琛. (2019). A Study on New Media Usage and Cultural Adaptation among Chinese Students in Contemporary Europe [当代中国留欧学生新媒体使用与文化适应研究]. *Journal of Overseas Chinese History Studies [华侨华人历史研究]*(01), 50-57.
73. Zhao [赵], W. 薇., & Li [李], Y. 越. (2019). An empirical study of Chinese first-degree students' Master's literacies and learning transitions in the UK [中国留英硕士生学术素养和身份转变研究]. *Tsinghua Journal of Education [清华大学教育研究], 40*(01), 79-86.
74. Zuo [左], L. 玲. (2016). Returning trends of overseas Chinese students in the early periods of the establishment of the People's Republic of China [新中国成立初期海外留学生群体的归国浪潮]. *Qinghai Social Sciences [青海社会科学]*(04), 190-197.

***English-language Articles (N=128)***

1. Ai, B. (2015). Living in-between: A narrative inquiry into the identity work of a Chinese student in Australia. *Life Writing, 12*(3), 353-368.
2. Bislev, A. (2017). Student-to-student diplomacy: Chinese international students as a soft-power tool. *Journal of Current Chinese Affairs, 46*(2), 81-109.
3. Cao, C., & Meng, Q. (2019). Mapping the paths from language proficiency to adaptation for Chinese students in a non-English speaking country: An integrative model of mediation. *Current Psychology, 38*(6), 1564-1575.
4. Cao, C., & Meng, Q. (2020). Effects of online and direct contact on Chinese international students’ social capital in intercultural networks: testing moderation of direct contact and mediation of global competence. *Higher Education*, 1-19.
5. Cao, C., Meng, Q., & Shang, L. (2018). How can Chinese international students’ host-national contact contribute to social connectedness, social support and reduced prejudice in the mainstream society? Testing a moderated mediation model. *International Journal of Intercultural Relations, 63*, 43-52.
6. Cao, C., Zhu, C., & Meng, Q. (2016). An exploratory study of inter-relationships of acculturative stressors among Chinese students from six European union (EU) countries. *International Journal of Intercultural Relations, 55*, 8-19.
7. Cao, L., & Tran, L. T. (2015). Pathway from vocational education and associate degree to higher education: Chinese international students in Australia. *Asia Pacific Journal of Education, 35*(2), 274-289.
8. Carnine, J. (2016). The Social Networks of Chinese Students Studying in France. *中国留学生在法国的社会关系., 12*(1), 69-95. doi:10.1163/17932548-12341317
9. Chen, H., Akpanudo, U., & Hasler, E. (2020). How Do Chinese International Students View Seeking Mental Health Services? *Journal of International Students, 10*(2), 286-305. doi:<https://doi.org/10.32674/jis.v10i2.765>
10. Chen, L. (2019). Problematising the English-only policy in EAP: a mixed-methods investigation of Chinese international students’ perspectives of academic language policy. *Journal of Multilingual and Multicultural Development*, 1-18.
11. Chen, P., You, X., & Chen, D. (2018). Mental Health and Cross-Cultural Adaptation of Chinese International College Students in a Thai University. *International Journal of Higher Education, 7*(4), 133-142.
12. Chen, Y., & Ross, H. (2015). “Creating a home away from home”: Chinese undergraduate student enclaves in US higher education. *Journal of Current Chinese Affairs, 44*(3), 155-181.
13. Cheng, P.-H., & Merrick, E. (2017). Cultural adaptation of dialectical behavior therapy for a Chinese international student with eating disorder and depression. *Clinical Case Studies, 16*(1), 42-57.
14. Choy, Y., & Alon, Z. (2019). The comprehensive mental health treatment of Chinese international students: a case report. *Journal of College Student Psychotherapy, 33*(1), 47-66.
15. Coates, J. (2019). The Cruel Optimism of Mobility: Aspiration, Belonging, and the “Good Life” among Transnational Chinese Migrants in Tokyo. *positions: asia critique, 27*(3), 469-497. doi:10.1215/10679847-7539277
16. Croucher, G., Zhong, Z., Moore, K., Chew, J., & Coates, H. (2019). Higher education student finance between China and Australia: Towards an international political economy analysis. *Journal of Higher Education Policy and Management, 41*(6), 585-599.
17. Curtin, K. D., Berry, T. R., & Walker, G. J. (2019). Older and More Experienced? Comparing Mainland Chinese International Students in Canada on Social Cognitive Correlates of Leisure Time Physical Activity, Acculturation, and Mental Health by Study Year. *Building Healthy Academic Communities Journal, 3*(2), 8-26.
18. Dai, K., & Hardy, I. (2020). The micro-politics of cultural change: a Chinese doctoral student’s learning journey in Australia. *Oxford Review of Education*, 1-17.
19. De Costa, P. I., Tigchelaar, M., & Cui, Y. (2016). Reflexivity and transnational habitus: The case of a ‘poor’affluent Chinese international student. *AILA Review, 29*(1), 173-198.
20. Ding, Q. (2016). Understanding Chinese International Doctoral Students in New Zealand: A Literature Review of Contemporary Writings about Chinese Overseas Research Students. *Teachers' Work, 13*(2), 118-133.
21. Ding, Q., & Devine, N. (2017). Agency and social capital in Chinese international doctoral students’ conversion to Christianity. *Educational Philosophy and Theory, 49*(12), 1161-1172.
22. Douglass, C. H., Qin, C., Martin, F., Xiao, Y., El-Hayek, C., & Lim, M. S. (2020). Comparing sexual behaviours and knowledge between domestic students and Chinese international students in Australia: findings from two cross-sectional studies. *International Journal of STD & AIDS, 0*(0), 1-10. doi:10.1177/0956462420921726
23. Du, Y., & Wei, M. (2015). Acculturation, enculturation, social connectedness, and subjective well-being among Chinese international students. *The Counseling Psychologist, 43*(2), 299-325.
24. Fan, Y.-S., & Maliborska, V. (2019). Birds of a Feather Flock Together?---A Case Study on Socialization Experiences of Chinese International Student in an American University. *International Journal of TESOL Studies*(2019-1), 43-70.
25. Fang, J., & Fine, G. A. (2020). Names and Selves: Transnational Identities and Self-Presentation among Elite Chinese International Students. *Qualitative sociology*. doi:10.1007/s11133-020-09468-7
26. Fong, M. (2019). Chinese International Students' Adaptive Orientations to Intercultural Compliment Interactions with Caucasian Americans. *China Media Research, 15*(3).
27. Fox, J. (2016). Common topic, similar hope: positioning of Chinese international students abroad. *MSU Working Papers in Second Language Studies, 7*(1), 6-23.
28. Fu, Y., Machado, C., & Weng, Z. (2018). Factors Influencing Chinese International Students’ Strategic Language Learning at Ten Universities in the US: A Mixed-Method Study. *Journal of International Students, 8*(4), 1891-1913.
29. Gao, H., Okoror, T. A., & Hyner, G. C. (2016). Focus group study of Chinese international students’ knowledge and beliefs about HPV vaccination, before and after reading an informational pamphlet about Gardasil. *Journal of immigrant and minority health, 18*(5), 1085-1092.
30. Gao, J. (2016). More Active Outside the Classroom Chinese International Students' Interactions with Chinese Communities in Australia [课堂以外更活跃：中国海外学生在澳洲与华人 社区的互动.]. *Journal of Chinese Overseas, 12*(1), 15-39. doi:10.1163/17932548-12341315
31. Gardiner, S., & Kwek, A. (2017). Chinese participation in adventure tourism: A study of generation Y international students’ perceptions. *Journal of Travel Research, 56*(4), 496-506.
32. Ge, L., Brown, D., & Durst, D. (2019). Chinese international students’ experiences in a Canadian university: Ethnographic inquiry with gender comparison. *Journal of International Students, 9*(2), 582-612.
33. Hail, H. C. (2015). Patriotism abroad: Overseas Chinese students’ encounters with criticisms of China. *Journal of Studies in International Education, 19*(4), 311-326.
34. Heng, T. T. (2017). Voices of Chinese international students in USA colleges:‘I want to tell them that…’. *Studies in higher education, 42*(5), 833-850.
35. Heng, T. T. (2019). Understanding the heterogeneity of international students’ experiences: A case study of Chinese international students in US universities. *Journal of Studies in International Education, 23*(5), 607-623.
36. Heng, T. T. (2020a). “Chinese Students Themselves Are Changing”. *Journal of International Students, 10*(2), 539-545.
37. Heng, T. T. (2020b). The Role of Theory in Qualitative Research: Insights from Studies on Chinese International Students in Higher Education. *Journal of International Students*. doi:<https://doi.org/10.32674/jis.v10i4.1571>
38. Hu, Y., van Veen, K., & Corda, A. (2016). Pushing too little, praising too much? Intercultural misunderstandings between a Chinese doctoral student and a Dutch supervisor. *Studying Teacher Education, 12*(1), 70-87.
39. Hu, Y., Xu, C. L., & Tu, M. (2020). Family-mediated migration infrastructure: Chinese international students and parents navigating (im)mobilities during the COVID-19 pandemic. *Chinese Sociological Review*, 1-26. doi:10.1080/21620555.2020.1838271
40. Jang, I. C., & Choi, L. J. (2020). Staying connected during COVID-19: The social and communicative role of an ethnic online community of Chinese international students in South Korea. *Miltilingua*, 1-12. doi:<https://doi.org/10.1515/multi-2020-0097>
41. Jensen, E. (2015). Creation of Third Space in a Bridge Class:'Dwelling In and Stretching Out'. *Case Studies Journal, 4*(11), 50-55.
42. Jia, F., & Koku, E. (2019). Music listening and cultural adaptation: How different languages of songs affect Chinese international students’ uses of music and cultural adaptation in the United States. *Journal of international and intercultural communication*, 1-18.
43. Kim, J. J., & Kim, I. (2019). Chinese international students’ psychological adaptation process in Korea: the role of tourism experience in the host country. *Asia Pacific Journal of Tourism Research, 24*(2), 150-167.
44. Kim, S. J., & Yoo, I. Y. (2016). Health promotion behavior of Chinese international students in Korea including acculturation factors: A structural equation model. *Asian nursing research, 10*(1), 25-31.
45. Kim, Y.-R. (2020). Classed education trajectories and intimate partnering of international students: a case of Chinese international undergraduate students in the United States. *Journal of Ethnic and Migration Studies*, 1-19. doi:10.1080/1369183X.2020.1828841
46. King, B., & Gardiner, S. (2015). Chinese international students. An avant‐garde of independent travellers? *International Journal of Tourism Research, 17*(2), 130-139.
47. Lai, H. (2015). Engagement and reflexivity: Approaches to Chinese-Japanese political relations by Chinese students in Japan. *Journal of Current Chinese Affairs, 44*(3), 183-212.
48. Lee, S. (2018). Frameworks for failure in L2 writing: What we can learn from “failures” of Chinese international students in the US. *Journal of Second Language Writing, 41*, 98-105.
49. Lee, S. W. (2017). Circulating East to East: Understanding the push–pull factors of Chinese students studying in Korea. *Journal of Studies in International Education, 21*(2), 170-190.
50. Lertora, I. M., & Sullivan, J. M. (2019). The Lived Experiences of Chinese International Students Preparing for the University-to-Work Transition: A Phenomenological Qualitative Study. *The Qualitative Report, 24*(8), COV5.
51. Li, G. (2016). Politically sensitive Chinese students’ engagement with democracy in Canada (对政治敏感的中国留学生在加拿大的民主参与：一个案例研究). *Journal of Chinese Overseas, 12*(1), 96-121. doi:doi:<http://dx.doi.org/10.1163/17932548-12341318>
52. Li, H., & Pitkänen, P. (2018). Understanding the Integration of Mainland Chinese Students: The Case of Finland. *Nordic Journal of Migration Research, 8*(2), 107-115.
53. Li, Z., Heath, M. A., Jackson, A. P., Allen, G., Fischer, L., & Chan, P. (2017). Acculturation experiences of Chinese international students who attend American universities. *Professional psychology: Research and practice, 48*(1), 11.
54. Lian, Z., & Wallace, B. C. (2020). Prevalence of past-year mental disorders and its correlates among Chinese international students in US higher education. *Journal of American college health, 68*(2), 176-184.
55. Lin, C., & Roy, S. (2019). English learning lived experiences of Chinese student newcomers in a Canadian postsecondary EAP programme: The role of gender. *The Asian Journal of Applied Linguistics, 6*(2), 197-209.
56. Liu, D. (2016). Strategies to promote Chinese international students’ school performance: resolving the challenges in American higher education. *Asian-Pacific journal of second and foreign language education, 1*(1), 1-15.
57. Liu, D., & Vogel, L. R. (2016). Mitigating Transitional Challenges of Chinese Students in US Higher Education. *Higher Education Studies, 6*(3), 100-113.
58. Liu, L. (2016). Chinese Student Migrants and American Religious Organizations. *Journal of Chinese Overseas, 12*(1), 122-153. doi:10.1163/17932548-12341319
59. Liu, N., & Zhang, Y. B. (2020). Warranting theory, stereotypes, and intercultural communication: US Americans’ perceptions of a target Chinese on Facebook. *International Journal of Intercultural Relations, 77*, 83-94.
60. Louie, A., & Qin, D. B. (2019). ‘Car Talk’: automobility and Chinese international students in Michigan. *Identities, 26*(2), 146-164.
61. Lu, H. (2015). Burgers or tofu? Eating between two worlds: Risk information seeking and processing during dietary acculturation. *Health communication, 30*(8), 758-771.
62. Lu, Y., Chui, H., Zhu, R., Zhao, H., Zhang, Y., Liao, J., & Miller, M. J. (2018). What does “good adjustment” mean for Chinese international students? A qualitative investigation. *The Counseling Psychologist, 46*(8), 979-1009.
63. Ma, J. (2017). Cooperative activity as mediation in the social adjustment of Chinese international students. *Journal of International Students, 7*(3), 856-875.
64. Ma, J. (2020). Supporting Practices to Break Chinese International Students' Language Barriers: The First Step to Facilitate Their Social Adjustment. *Journal of International Students, 10*(1), 84-105.
65. Ma, T., Heywood, A., & MacIntyre, C. R. (2020). Travel health risk perceptions of Chinese international students in Australia – Implications for COVID-19. *Infection, Disease & Health*. doi:<https://doi.org/10.1016/j.idh.2020.03.002>
66. Maeder-Qian, J. (2017). Linguistic identity changes of Chinese international students in Germany: A pilot study. *Study Abroad Research in Second Language Acquisition and International Education, 2*(2), 240-262.
67. Martin, F. (2017a). Mobile self-fashioning and gendered risk: rethinking Chinese students’ motivations for overseas education. *Globalisation, Societies and Education, 15*(5), 706-720. doi:10.1080/14767724.2016.1264291
68. Martin, F. (2017b). Rethinking network capital: hospitality work and parallel trading among Chinese students in Melbourne. *Mobilities, 12*(6), 890-907.
69. Martin, F. (2020). Iphones and “African gangs”: everyday racism and ethno-transnational media in Melbourne’s Chinese student world. *Ethnic and Racial Studies, 43*(5), 892-910. doi:10.1080/01419870.2018.1560110
70. McCrohon, M., & Nyland, B. (2018). The perceptions of commoditisation and internationalisation of higher education in Australia: An interview study of Chinese international students and their lecturers. *Asia Pacific Education Review, 19*(1), 17-26.
71. Meng, Q., Li, J., & Zhu, C. (2019). Towards an ecological understanding of Chinese international students’ intercultural interactions in multicultural contexts: Friendships, inhibiting factors and effects on global competence. *Current Psychology*, 1-14.
72. Meng, Q., Zhu, C., & Cao, C. (2017). The role of intergroup contact and acculturation strategies in developing Chinese international students’ global competence. *Journal of Intercultural communIcatIon research, 46*(3), 210-226.
73. Meng, Q., Zhu, C., & Cao, C. (2018). Chinese international students’ social connectedness, social and academic adaptation: The mediating role of global competence. *Higher Education, 75*(1), 131-147.
74. Montalbano, R., Creghan, C., Eidson, K., & Maninger, R. M. (2016). Chinese Students and the American Dream: Are These Students Prepared for Success? *American International Journal of Humanities and Social Science, 2*(3), 15-25.
75. Montsion, J.-M. (2020). Making Sense of One’s Feelings: The Emotional Labour of Chinese International Students in Canadian Universities. *Migration, Mobility, & Displacement, 5*(1), 3-19.
76. Okura, K. (2019). There are no Asians in China: the racialization of Chinese international students in the United States. *Identities*, 1-19.
77. Qi, W. G., Wang, Z., Wu, L. Z., & Luo, X. (2019). Multicultural supervision with Chinese international trainees. *Training and Education in Professional Psychology, 13*(3), 185.
78. Redfern, K. (2016). An empirical investigation of the incidence of negative psychological symptoms among Chinese international students at an Australian university. *Australian Journal of Psychology, 68*(4), 281-289.
79. Ross, H., & Chen, Y. (2015). Engaging Chinese international undergraduate students in the American university. *Learning and Teaching, 8*(3), 13-36.
80. Sato, T., Burge-Hall, V., & Matsumoto, T. (2020). American Undergraduate Students’ Social Experiences With Chinese International Students. *International Journal of Educational Reform*, 1056787920927682.
81. Scally, J., & Jiang, M. (2019). ‘I wish I knew how to socialize with native speakers’: supporting authentic linguistic and cultural experiences for Chinese TESOL students in the UK. *Journal of Further and Higher Education*, 1-14.
82. Song, X. (2019). ‘Chinese Students Syndrome’ in Australia: colonial modernity and the possibilities of alternative framing. *Higher Education*, 1-14.
83. Su, M., & Harrison, L. M. (2016). Being wholesaled: An investigation of Chinese international students’ higher education experiences. *Journal of International Students, 6*(4), 905-919.
84. Sude, Yuan, M., Chen, N., & Dervin, F. (2020). “I think it would be easier for Chinese ethnic minorities to find themselves as a minority if they go abroad”: Chinese Minzu individuals’ identity and the study abroad experience. *International Journal of Educational Research, 102*. doi:<https://doi.org/10.1016/j.ijer.2020.101584>
85. Sun, Q., Nguyen, T. D., & Ganesh, G. (2020). Exploring the Study Abroad Journey: Chinese and Indian Students in US Higher Education. *Journal of International Consumer Marketing, 32*(3), 210-227.
86. Sun, X., & Rhoads, R. A. (2018). Chinese University Students and Their Experiences of Acculturation at an Ethnic Christian Church. *Journal of International Students, 8*(1), 131–150-131–150.
87. Suspitsyna, T., & Shalka, T. R. (2019). The Chinese International Student as a (Post) Colonial Other: An Analysis of Cultural Representations of a US Media Discourse. *The Review of Higher Education, 42*(5), 287-308.
88. Tsai, P.-C., & Wei, M. (2018). Racial discrimination and experience of new possibilities among Chinese international students. *The Counseling Psychologist, 46*(3), 351-378.
89. Tsai, W., Wang, K. T., & Wei, M. (2017). Reciprocal relations between social self-efficacy and loneliness among Chinese international students. *Asian American Journal of Psychology, 8*(2), 94.
90. Tu, M. (2016). Chinese one-child families in the age of migration: Middle-class transnational mobility, ageing parents, and the changing role of filial piety. *The Journal of Chinese Sociology, 3*(1), 1-17. doi:10.1186/s40711-016-0036-z
91. Tu, M., & Nehring, D. (2019). Remain, Return, or Re-migrate? The (Im)mobility Trajectory of Mainland Chinese Students after Completing Their Education in the UK. *International Migration, 58*(3), 43-57. doi:10.1111/imig.12589
92. Tu, M., & Xie, K. (2020). Privileged Daughters? Gendered Mobility among Highly Educated Chinese Female Migrants in the UK. *Social Inclusion, 8*(2), 68-76. doi:10.17645/si.v8i2.2675
93. Valdez, G. (2015). US higher education classroom experiences of undergraduate Chinese international students. *Journal of International Students, 5*(2), 188-200.
94. Wang, K. T., Tian, L., Fujiki, M., & Bordon, J. J. (2017). Do Chinese International Students’ Personalities Change During Cross-National Transitions? *Journal of International Students, 7*(2), 229-245.
95. Wang, X. (2017). Spatial and literacy practices of Chinese international students across a bridge writing classroom and WeChat. *Language and Education, 31*(6), 561-579.
96. Weng, T.-h. (2020). On becoming a doctoral student: Chinese doctoral students’ socialization of capital and habitus in academia. *British Journal of Sociology of Education*, 1-19. doi:10.1080/01425692.2020.1745056
97. Will, N. L. (2017). The Influx of Chinese International Students and Impact on US Private Secondary Schools. *International Journal of Arts Humanities and Social Sciences, 2*(3), 1-9.
98. Will, N. L. (2019). From isolation to inclusion: Learning of the experiences of Chinese international students in US. *Journal of International Students, 6*(4), 1069-1075.
99. Wu, B. (2016). Chinese Student Migration, Social Networking, and Local Engagement in the uk (华人留学生移民, 社交网络与参与当地社会: 一个基于英国的实证研究): An Empirical Study. *Journal of Chinese Overseas, 12*(1), 40-67.
100. Wu, S., Wu, Q., Wei, X., Bledsoe, S. E., & Ansong, D. (2020). Exploring Factors for Achieving Successful Educational Attainment among Chinese Doctoral Students in the United States. *Journal of International Students, 10*(2), 244-264.
101. Wu, X. (2020a). Examining the influence of transnational discourses on Chinese international secondary school students’ academic learning. *Journal of Multilingual and Multicultural Development, 41*(4), 368-382.
102. Wu, X. (2020b). Reflexivity in multilingual and intercultural education: Chinese international secondary school students’ critical thinking. *Journal of Multilingual and Multicultural Development*, 1-15.
103. Wu, X., & Tarc, P. (2019). Chinese international students in a Canadian private secondary school: becoming flexible citizens? *Compare: A Journal of Comparative and International Education*, 1-19. doi:10.1080/03057925.2019.1684242
104. Wu, Y. (2020). Study Abroad Experience and Career Decision-Making: A Qualitative Study of Chinese Students. *Frontiers of Education in China, 15*(2), 313-331. doi:10.1007/s11516-020-0014-8
105. Xu, C. L. (2021). Time, class and privilege in career imagination: Exploring study-to-work transition of Chinese international students in UK universities through a Bourdieusian lens. *Time & Society, 30*(1), 5-29. doi:10.1177/0961463x20951333
106. Xu, C. L., & Montgomery, C. (2019). Educating China on the Move: A Typology of Contemporary Chinese Higher Education Mobilities. *Review of Education, 7*(3), 598–627. doi:<https://doi.org/10.1002/rev3.3139>
107. Xu, H., O'Brien, W. H., & Chen, Y. (2020). Chinese international student stress and coping: A pilot study of acceptance and commitment therapy. *Journal of Contextual Behavioral Science, 15*, 135-141.
108. Xu, X., Sit, H., & Chen, S. (2020). International education through a bioecological development lens – a case study of Chinese doctoral students in Australia. *Higher Education Research & Development*, 1-16. doi:10.1080/07294360.2020.1811646
109. Yan, Z., Cardinal, B. J., & Acock, A. C. (2015). Understanding Chinese international college and university students' physical activity behavior. *Journal of Sport and Health Science, 4*(2), 203-210.
110. Yao, C. W. (2016a). " Better English is the Better Mind": Influence of Language Skills on Sense of Belonging in Chinese International Students. *Journal of College & University Student Housing, 43*(1).
111. Yao, C. W. (2016b). Unfulfilled expectations: Influence of Chinese international students’ roommate relationships on sense of belonging. *Journal of International Students, 6*(3), 762-778.
112. Yao, C. W. (2018). " They Don't Care About You": First-Year Chinese International Students' Experiences With Neo-racism and Othering on a US Campus. *Journal of The First-Year Experience & Students in Transition, 30*(1), 87-101.
113. Yu, Y. (2019). From universities to Christian churches: agency in the intercultural engagement of non-Christian Chinese students in the UK. *Higher Education*. doi:10.1007/s10734-019-00474-5
114. Yu, Y., & Moskal, M. (2019a). Missing intercultural engagements in the university experiences of Chinese international students in the UK. *Compare: A Journal of Comparative and International Education, 49*(4), 654-671.
115. Yu, Y., & Moskal, M. (2019b). Why do christian churches, and not universities, facilitate intercultural engagement for Chinese international students? *International Journal of Intercultural Relations, 68*, 1-12. doi:<https://doi.org/10.1016/j.ijintrel.2018.10.006>
116. Zhang-Wu, Q. (2018). Chinese International students’ experiences in American higher education institutes: A critical review of the literature. *Journal of International Students, 8*(2), 1173-1197.
117. Zhang, F., & Zhan, J. (2020). Understanding voice in Chinese students’ English writing. *Journal of English for Academic Purposes, 45*, 100844.
118. Zhang, Q. (2016). Barriers and Strategies: How do Chinese international students navigate between modern western biomedicine and traditional Chinese medicine? *International Journal of Arts & Sciences, 9*(2), 357.
119. Zhang, S., & Xu, C. L. (2020). The making of transnational distinction: an embodied cultural capital perspective on Chinese women students’ mobility. *British Journal of Sociology of Education, 41*(8), 1251-1267. doi:10.1080/01425692.2020.1804836
120. Zhang, X. (2018). Chinese international students in New Zealand: Views of citizenship and democracy. *Citizenship Teaching & Learning, 13*(2), 241-252.
121. Zhang, Y., & Jung, E. (2017). Multi-Dimensionality of Acculturative Stress among Chinese International Students: What Lies behind Their Struggles? *International Research and Review, 7*(1), 23-43.
122. Zhang, Z. O., & Beck, K. (2017). Seeking Sanctuary: Chinese Student Experiences of Mobility and English Language Learning in Canada. *International Journal of Chinese Education, 6*(2), 176-209.
123. Zhao, X. (2019). Disconnective intimacies through social media: practices of transnational family among overseas Chinese students in Australia. *Media International Australia*. doi:.org/10.1177/1329878X19837684
124. Zhao, X. (2020). Digital labour in transnational mobility: Chinese international students’ online boundary work in daigou. *New Media & Society, 0*(0), 1461444820934096. doi:10.1177/1461444820934096
125. Zheng, K., & West-Olatunji, C. A. (2016). Mental Health Concerns of Mainland Chinese International Students in the United States: A Literature Review. *VISTAS Online*, 1-12.
126. Zhu, Y. (2019). Social media engagement and Chinese international student recruitment: understanding how UK HEIs use Weibo and WeChat. *Journal of Marketing for Higher Education, 29*(2), 173-190.
127. Zhu, Y., & Bresnahan, M. (2018). They make no contribution!” versus “We should make friends with them!”—American domestic students’ perception of Chinese international students' reticence and face. *Journal of International Students, 8*(4), 1614-1635.
128. Zhu, Y., & Bresnahan, M. J. (2018). Collective face, politeness strategies, and discomfort: Communication of American domestic students and Chinese international students. *Journal of Intercultural communIcatIon research, 47*(2), 141-159.

**Appendix 2. Subject positions and articles reviewed**

| Subject Position | Sub-categories | Articles reviewed |
| --- | --- | --- |
| 1. Neoliberal   (N=48: N=12 Chinese-language + N=36 English-language) | 1. Cash cows 2. Embedded in global neoliberal machinery, contributing to reproducing global inequalities, but also as victims 3. Individualised without individuality vis-a-vis the Chinese state; responsibilised for own successes or failures, instrumentalised as means for achieving China’s modernisation; enterprising, calculating, striving 4. ‘Side effect’: suffering from mental health issues | Chen & Ross, 2015; Ding, 2016; Ding & Devine, 2017; Fang & Fine, 2020; Fox, 2016; Gao, 2016; Ross & Chen, 2015; Cao, Meng, & Shang, 2018; Chen [陈], 2015; Chen, Akpanudo, & Hasler, 2020; Chen, You, & Chen, 2018; Choy & Alon, 2019; Croucher et al., 2019; Jang & Choi, 2020; Jiang [姜] & Jiang [蒋], 2017; Jin [金], 2015; Kim & Yoo, 2016; Kuang [旷] & Qi [戚], 2016; Lee, 2017; Lertora & Sullivan, 2019; Lian & Wallace, 2020; Linghu [令狐], 2019; Louie & Qin, 2019; Ma, Heywood, & MacIntyre, 2020; McCrohon & Nyland, 2018; Su & Harrison, 2016, p. 907; Sun et al., 2020; Tao [陶] & Liu [刘], 2016; Tu [屠] & He [何], 2020; Tu & Nehring, 2019; Tu & Xie, 2020; Wu [吴] & Huang [黄], 2017; Wu, 2020; Wu & Tarc, 2019; Wu, 2020b; Xiao [肖], 2015; Xu, 2021; Xu and Montgomery, 2019; Xu, O'Brien, & Chen, 2020; Yao, 2016a, 2016b; Ye [叶] & Wang [王], 2018; Yu, 2019; Zhai [翟] & Gao [高], 2018; Zhang [张], 2018; Zhang & Beck, 2017; Zhang [章] & Hu [胡], 2019; Zheng & West-Olatunji, 2016 |
| 1. Political   (N=52: N=39 Chinese-language + N=13 English-language) | 1. Instruments of states (both Chinese and foreign)   A1: strategic human capital for China’s modernisation  A2: cultural ambassadors or people-to-people diplomats  A3: potential spies and political/diplomatic sacrifices   1. Ethno-national 2. Ideological   C1: Ideological mediators  C2: negotiating what democracy means and how it works  C3: malleable but also suspicious | Bislev, 2017; Carnine, 2016; Chen [陈], 2018; Chen [陈], 2018, 2019; Chen [陈], 2015; Cheng & Merrick, 2017; Chu [楚] & Zhang [张], 2020; Cui [崔], 2018; Fan & Maliborska, 2019; Fong, 2019; Fu [付], 2018; Gan [干] & Liu [刘], 2016; Gao [高], 2017; Hail, 2015; Hao [郝], 2016; Heng, 2017; Hu [胡], 2016; Hu [胡], 2015; Hu [胡], 2018; Jin [金], 2015; Jing [荆], 2015; Lai, 2015; Li [李], 2016; Li [李] & Sun [孙], 2019; Li, 2016; Li & Pitkänen, 2018; Lin [林], 2016, 2017a, 2017b; Linghu [令狐], 2019; Liu [刘] & Yu [余], 2015; Liu [柳], 2016; Maeder-Qian, 2017; Ren [任] & Liang [梁], 2020; Song, 2019; Wang [王] & Ding [丁], 2020; Wang [王] & Zhang [张], 2018; Wang [王], 2016; Wang [王], 2020; Wu [吴] & Huang [黄], 2017; X. Wu & Tarc, 2019; Xu [徐], 2020; Ye [叶] & Wang [王], 2018; Yuan[元] & Yue [岳], 2015; Zhang [张], 2018; Zhang [张], 2019; Zhang, 2016; Zhang, 2018; Zhao, 2019; Zuo [左], 2016 |
| 1. Pedagogic   (N=38: N=4 Chinese-language; N=34 English-language) | 1. Deficient, incompetent, reticent, lacking critical thinking 2. Knowledgeable educators 3. Agentic, methodical, resourceful, innovative | Chen & Ross, 2015; Fox, 2016; Jensen, 2015; Heng, 2017, 2019; Zhang & Beck, 2017; Liu, 2016; Liu & Vogel, 2016; Ma, 2017, 2020; Zhang & Jung, 2017; Zhao [赵] & Li [李], 2019; Chen [陈], 2018; Chen, 2019; Dai & Hardy, 2020; De Costa, Tigchelaar, & Cui, 2016; Fan & Maliborska, 2019; Fu, Machado, & Weng, 2018; Gao, Okoror, & Hyner, 2016; Hu [胡], 2016; Hu, van Veen, & Corda, 2016; Kim & Kim, 2019; Lee, 2018; Li & Pitkänen, 2018; Lin & Roy, 2019; McCrohon & Nyland, 2018; Qi [齐], 2017; Sato, Burge-Hall, & Matsumoto, 2020; Scally & Jiang, 2019; Valdez, 2015; Wang, 2017; Weng, 2020; Will, 2017; Wu, 2020a, 2020b; Xu, 2021; Xu, Sit, & Chen, 2020; Zhang & Zhan, 2020; Zhu & Bresnahan, 2018a, 2018b. |
| 1. Racialised   (N=25: N=6 Chinese-language + N=19 English-language) | 1. Racialised   A1: Discrimination considered as racism, no further discussion  A2: Postcolonial critiques & in-depth depiction of racialised experiences   1. Ethnically diverse within China | Cang [苍] & Zheng [郑], 2016; Chen, 2019; Fang & Fine, 2020; Hao [郝], 2016; Hu, Xu & Tu, 2020; Kuang [旷] & Qi [戚], 2016; Lin [林], 2016; Lin [林] & Chen [陈], 2019; Louie & Qin, 2019; Lu, 2015; Lu et al., 2018; Meng, Li, & Zhu, 2019; Okura, 2019; Sude, Yuan, Chen, & Dervin, 2020; Suspitsyna & Shalka, 2019; Tsai & Wei, 2018; Valdez, 2015; Will, 2019; Wu, 2016; Xu, 2021; Yan, Cardinal, & Acock, 2015; Yao, 2018; Zhang-Wu, 2018; Zhang [张], 2016; Zhang & Jung, 2017; Zhu & Bresnahan, 2018a, 2018b |
